# Coping and Caregiving Experiences Among Siblings of Individuals with Severe Mental Disorders

**DOI:** 10.3390/healthcare14030388

**Published:** 2026-02-03

**Authors:** Carolina Reyes-González, Mª Nieves Pérez-Marfil, Isabel C. Salazar

**Affiliations:** 1 Independent Researcher, 18071 Granada, Spain; ps.carolinareyesgonzalez@gmail.com; 2 Department of Personality, Assessment and Psychological Treatment, Mind, Brain and Behavior Research Center (CIMCYC), University of Granada, 18071 Granada, Spain; nperez@ugr.es

**Keywords:** coping strategies, severe mental disorder, siblings, informal caregivers, perceived health, perceived stress, self-esteem, caregiving experience

## Abstract

**Background/Objective:** Informal caregiving for individuals with severe mental disorders (SMDs) often leads to significant psychological distress. However, the specific coping strategies that determine mental health outcomes among siblings remain poorly understood. This study aimed to analyze the predictive capacity of various coping strategies regarding health, perceived stress, self-esteem, and caregiving experience for siblings. **Methods:** A cross-sectional study was conducted with a sample of siblings of patients with SMDs (*N* = 60) from mental health service. Self-report measures were used to assess perceived health, perceived stress, self-esteem, coping strategies, and caregiving experience. Multiple linear regression analyses were performed for each dependent variable, controlling for collinearity. **Results:** The siblings reported a higher mean use of problem-focused coping strategies compared to emotion-focused coping strategies. Regression models were statistically significant for all analyzed variables, except for somatic symptoms. Emotion-focused maladaptive coping (EFMC) strategies emerged as the most consistent and powerful predictor, showing a significant association with positive caregiving appraisal (β = 0.657), depression (β = 0.500), poor health (β = 0.453), negative stress (β = 0.449), social dysfunction (β = 0.429), self-esteem (β = −0.390), and anxiety (β = 0.368). In contrast, problem-focused strategies were largely non-significant, except for an association with positive and negative aspects of caregiving (β_PFMC_ = 0.509, β_PFMC_ = 0.312, respectively), and positive stress (β_PFAC_ = −0.272). **Conclusions:** These results suggest that while siblings of people with SMDs report a greater use of problem-focused coping strategies, the adoption of EFMC strategies is the most detrimental factor observed, given their negative influence on mental health, self-esteem, and caregiving experience.

## 1. Introduction

Severe mental disorders (SMDs), such as schizophrenia, bipolar disorder, borderline personality disorder, and major depressive disorder, have a significant impact on patients’ lives and family dynamics [1]. This phenomenon’s impact extends beyond the immediate patients, resulting in significant consequences for informal caregivers (e.g., family members, partners, friends) [2]. In the field of mental health, parents are the most frequent caregivers.

Providing care for individuals with SMDs appears to have negative effects on the physical and emotional health of informal caregivers. Some studies report the presence of symptoms of anxiety and depression, elevated levels of stress, and lower life satisfaction [3,4,5]. Family members and informal caregivers also experience a significant deterioration in their physical and emotional well-being associated with chronic stress, caregiving overload, and lack of adequate support, which increases the risk of physical and mental health problems, particularly distress, anxiety, and depression [6]. In informal caregivers of people with schizophrenia, a high emotional burden and substantial deterioration in physical and mental health have been reported [7]. Prolonged informal care also negatively affects caregivers’ self-esteem, especially if they perceive that they are not meeting social or family expectations, often experiencing a sense of personal failure in the face of their loved ones’ deterioration [8].

Clinical symptoms and self-esteem issues appear to be related to caregiver overload, which, in turn, negatively impacts the ability to cope with the diagnosis [9,10]. Several studies have demonstrated the importance of coping strategies for the well-being of family members with SMDs [11,12,13]. Maladaptive coping strategies, such as avoidance, denial, and concealment of the problem, are more common in stressful situations related to mental disorders [14]. Avoidance and resignation appear to aggravate the mental health of family members, while problem-focused adaptive coping promotes positive long-term outcomes [15]. Likewise, the use of strategies such as seeking social support reduces stress levels in informal caregivers of patients with psychotic disorders [16], and it is believed that those who perceive social support experience less emotional overload [12]. Thus, emotional and physical overload is a critical factor in the experience of family caregivers [17].

The experience of siblings of patients with SMDs differs from that of other family members, such as parents, due to relational, developmental, and practical factors that shape their caregiving experience. The sibling relationship has unique characteristics that influence how they cope with SMDs, including difficulties in sharing emotions with other family members and greater indirect emotional involvement, which can lead to feelings of sadness, fear, anger, and confusion [1,5,18,19]. From a personal development perspective, siblings must adjust to new roles within the home, such as taking on caregiving responsibilities, while their emotional development is affected by prolonged exposure to family stress and managing the patient’s symptoms, which can lead to social isolation and difficulties in coping with complex situations [5,20,21,22,23,24]. On a practical level, siblings seem to have difficulties related to the use of maladaptive coping strategies, such as avoidance, escape, and isolation, and to the absence of psychoeducation interventions that could improve their knowledge of the disorder, alleviate the emotional burden, and better manage stress [1,5,21,22]. Taken together, these conditions may influence siblings’ experiences to be different from those of parents. Therefore, studies are needed that consider the unique role of siblings within the family system and their emotional well-being in the context of SMDs [21,22,25,26,27].

The literature on the experiences of siblings of those with SMDs is generally scarce. Evidence indicates that the diagnosis has a unique and significant impact on siblings’ lives [18]. Sibling dynamics can become complex as they attempt to understand and manage the symptoms and behaviors associated with mental illness [27]. Siblings often isolate themselves [23], have difficulty coping with the diagnosis (and what it entails) [5], and perceive the experience as a great challenge [1]. Factors such as the role assumed in the family, the burden of care [28], the quality of the previous relationship [22], the patient’s symptoms [18], and the stress generated by the situation [5] significantly influence the siblings’ experience.

After knowing of the diagnosis, siblings face specific challenges: Difficulty understanding the disorder, adjusting to new family roles (including the role of caregiver), and managing possible stigmatizing reactions [22]. Some authors point out that siblings are not usually the primary caregivers, but they do take on significant emotional and practical support responsibilities, particularly when parents face limitations or the patient requires continuous supervision [18,22]. Thus, the experience of having a sibling with an SMD can affect their quality of life [21,22,28,29]. The literature on this subject is also scarce in other countries, making cultural comparisons difficult. For example, in the Latin American context, informal care for people with severe mental illnesses is concentrated among mothers and wives as primary caregivers; however, siblings also play a significant role as secondary caregivers. Their involvement is expressed through emotional support, the distribution of practical tasks, and financial assistance, especially when primary caregivers face exhaustion or limitations. Although their role is less visible in public policies, the recent literature acknowledges that the participation of siblings contributes to family resilience and the sustainability of care, partially relieving the burden of those who assume the main responsibility [30].

Considering the above, studying siblings specifically allows us to understand and address their particular needs, preventing their experience from being subsumed within general research on family caregivers and offering an innovative and targeted approach. Therefore, this study aims to identify the coping strategies employed by the siblings of people with SMDs and assess how they relate to their health, perceived stress, self-esteem and the experience of caring for their siblings. We hypothesize that different coping strategies (or styles) will be utilized and that maladaptive strategies will be significantly associated with poorer perceived health, higher perceived stress levels, lower self-esteem, and a poorer assessment of the caregiving experience for patients with SMDs.

## 2. Materials and Methods

### 2.1. Study Design

This is a cross-sectional observational study.

### 2.2. Sample

This study utilized a non-probability convenience sampling. Mental health professionals initially identified eligible patients who had siblings. Subsequently, permission was requested from the patients to invite their siblings to participate in the study. Of the 101 siblings initially contacted, 41 declined to participate. Reasons cited for non-participation were lack of closeness to the patient, not being involved in the patient’s care, or the belief that care responsibility belonged to the parents. The final sample of 60 siblings of patients with SMDs, treated at three mental health units at the Virgen de las Nieves University Hospital in Granada, Spain, included 37 women and 23 men. Their ages ranged from 18 to 61 years (*M* = 36.4, *SD* = 12.1).

The inclusion criteria were: Being the sibling of a patient diagnosed with SMD, living with and/or participating in the informal care of the patient with SMD (at least one day per month), committing the necessary time (1–2 h) or the psychological evaluation, and agreeing to participate in the study and sign the informed consent form. The exclusion criteria were: Having a diagnosis of SMD or neurological disease, brain damage, substance abuse/dependence, intellectual disability or having received any type of care/support from a mental health unit in the past.

Table 1 shows the main sociodemographic and contextual characteristics of the 60 siblings. Participants were between 18 and 61 years old (*M* = 36.4, *SD* = 12.1) and were mostly female (61.7%). More than half were single (56.6%) and had a high school education or higher (86.9%). Most were employed (53.3%) or students (26.6%), and a minority were unemployed (14.9%) or homemakers (5%).

### 2.3. Instruments

#### 2.3.1. The Ad Hoc Personal Information Questionnaire

An ad hoc personal information questionnaire collects information on age, gender, current marital status, level of education, employment status, prior therapeutic history for the patient’s diagnosis, whether they are aware of the diagnostic label assigned to their sibling, whether they are the patient’s primary and/or sole caregiver, and whether they live in the same household as the patient.

#### 2.3.2. The Spanish Version of the Coping Strategies Inventory (CSI)

The CSI consists of 40 items and assesses eight coping strategies that are grouped into four categories or coping styles: Problem-focused adaptive coping (PFAC) strategies (problem solving and cognitive restructuring), emotion-focused adaptive coping (EFAC) strategies (social support and express emotions), problem-focused maladaptive coping (PFMC) strategies (problem avoidance and wishful thinking), and emotion-focused maladaptive coping (EFMC) strategies (social withdrawal and self-criticism) [31,32]. The items are answered on a five-point Likert scale. A high score indicates greater use of the strategies and a coping style. The reported internal consistency (Cronbach’s alpha) is between 0.63 and 0.86 [31]. In the present study, Cronbach’s alpha for the total CSI score was 0.78. Reliability coefficients for the subscales were as follows: Problem solving (0.86), cognitive restructuring (0.80), social support (0.80), express emotion (0.84), problem avoidance (0.63), wishful thinking (0.78), social withdrawal (0.65), and self-criticism (0.89).

#### 2.3.3. The Spanish Version of the General Health Questionnaire (GHQ-28)

The GHQ-28 consists of 28 items that assess overall health perception and potential current psychopathology [33,34]. It further differentiates health perception into four subscales: Physical symptoms, anxiety/anguish, social dysfunction, and depression. The items have four response options, the first two scored as 0 and the last two as 1, yielding a maximum overall score of 28 [33]. Higher scores indicate a greater level of psychological distress in each area. The reported reliability (Cronbach’s alpha) for the total scale score is 0.97 [35]. In the present study, Cronbach’s alpha for the total GHQ-28 score was 0.95. Subscale reliability coefficients were as follows: Somatic symptoms (0.85), anxiety/insomnia (0.86), social dysfunction (0.82), and severe depression (0.89).

#### 2.3.4. The Spanish Version of the Rosenberg Self-Esteem Scale (RSES)

The RSES consists of 10 items with four-point Likert scale response options [36,37]. Higher scores on the RSES indicate greater self-esteem. The interpretation of the Spanish version uses the following criteria: 30–40 points for high self-esteem; 26–29 points for normal self-esteem; and scores below 26 points for low self-esteem. The RSES reports an internal consistency (Cronbach’s alpha) of 0.87 [37] and it was 0.82 in this study.

#### 2.3.5. The Spanish Version of the Perceived Stress Scale (PSS)

The PSS consists of 14 items that assess the level of stress perceived during the last month [38,39]. The response options utilize a five-point Likert scale. A high total score indicates a higher level of perceived stress; however, items 4, 5, 6, 7, 9, and 10 must be reverse-scored prior to calculating the final score to ensure consistent polarity across the scale. Interpretation is based on the following criteria: 0 to 14, low stress; 15 to 23, occasionally stressed; 24 to 42, often stressed; and 43 to 56, very often stressed on the total score [39]. In our study, we did not employ the total score; instead, we utilized two subscales: Positive stress (reflecting perception of control and coping ability; items 4, 5, 6, 7, 9 and 10) and negative stress (reflecting perception of stress, worry, inability to control situations; items 1, 2, 3, 8, 11 and 12). As the positively worded items were reverse-scored –in accordance with the original scoring procedure–, their interpretation was conducted such that lower scores indicate greater perceived control and stronger coping resources, whereas higher scores reflect reduced perceived control and coping capacity. The Spanish version of the PSS has demonstrated adequate internal consistency (α = 0.82) [39]. In the present study, reliability estimates (Cronbach’s alpha) were 0.82 for the total score, 0.80 for the positively worded items subscale, and 0.81 for the negative perceived stress subscale.

#### 2.3.6. The Spanish Version of the Caregiving Inventory (ECI)

The ECI consists of 66 items and assesses positive and negative perceptions of patient care [40,41]. Positive aspects are grouped into two factors: Satisfying personal experiences of care and positive aspects of the relationship with the patient. Negative aspects include eight factors: Difficult behaviors, negative symptoms, stigma, problems with social and health services, consequences for the family, need for support, dependence, and loss. The items are scored on a four-point Likert scale. A high score indicates positive and negative subjective appraisal of care, respectively. The reported Cronbach’s alpha is between 0.72 and 0.88 [40]. In the present study, Cronbach’s alpha was 0.90 for the total ECI score Reliability for the subscales was as follows: Difficult behaviors (0.76), negative symptoms (0.78), stigma (0.77), problems with services (0.74), effects on the family (0.76), need for backup (0.77), dependency (0.77), loss (0.77), satisfactory personal experiences (0.82), and positive aspects of the relationship (0.82).

### 2.4. Procedure

The study was approved by the mental health service management, the coordinating bodies of the mental health units, and the Hospital Ethics Committee (code 1894-N-19) and followed the principles of the Declaration of Helsinki. Permission was requested from the individuals diagnosed with an SMD to contact their siblings, who were informed about the objectives of the study and signed the informed consent form. The participation of the siblings was voluntary. Data was collected in three mental health units with the collaboration of the clinical teams. The assessments were conducted individually at the MHU in 60–90-min sessions. The order of administration of the instruments was: Personal Information Form, RSES, CSI, GHQ-28, PSS, and ECI.

### 2.5. Data Analysis

Data analysis was performed using the JASP v. 0.16.3 software program [42]. Prior to descriptive and inferential analyses, compliance with the statistical assumptions of normality was assessed using the Kolmogorov–Smirnov test, and homoscedasticity was assessed using Levene’s test. Descriptive statistics (frequencies, means, and standard deviations) were calculated to characterize the sample and the main variables of the study.

A repeated-measures ANOVA was performed to compare the relative frequency of the four coping strategies within the participant group. Since each sibling provided scores for all strategies, a dependent-measures design was required. This approach is suitable for analyzing continuous variables collected from a single group during the same assessment. Prior to the ANOVA, the assumption of sphericity was tested, and the Greenhouse-Geisser correction was applied where necessary. Post hoc comparisons with Bonferroni correction were performed to identify specific differences between coping strategies.

Pearson correlations (*r*) were then calculated to explore associations between variables. Subsequently, multiple linear regression models were then constructed to evaluate coping strategies as predictors of several psychological variables (somatic symptoms, anxiety/anguish, social dysfunction, depression, overall health, positive stress, negative stress, self-esteem, and caregiving appraisal). To ensure model stability and prevent overfitting with the available sample size (*n* = 60), a simultaneous entry method was used without covariates. Multicollinearity was monitored through variance inflation factor (VIF) (VIF = 1 absent; 1 ≤ VIF ≤ 5 moderate) and tolerance (T) (T ≥ 0.20 acceptable; T = 1 absent) statistics. The reliability and validity of the regression model were examined by analyzing the model’s assumptions through the residuals and the identification of influential observations. The assumption of homoscedasticity (constant error variance) was assessed by inspecting the scatter plot of the Residuals versus Predicted Values. A random distribution of the residuals around the zero line will indicate compliance with this assumption. The normality of the errors will be checked visually using a Q-Q Plot of the Standardized Residuals; a close alignment of the points to the diagonal reference line will support the normality assumption. Finally, to evaluate the impact of each observation on the model coefficients, Cook’s Distance was calculated and analyzed. Cases exhibiting a significantly high Cook’s Distance will be closely examined to determine if they represent outliers or exert undue influence on the model’s results.

## 3. Results

### 3.1. Descriptive Results

Before conducting descriptive and inferential analyses, compliance with the assumptions of normality and homoscedasticity was verified. The results of the Kolmogorov–Smirnov test indicated that the distributions of the main variables followed a normal distribution (*p* > 0.05). Similarly, Levene’s test confirmed the equality of variances between groups (homoscedasticity) (*p* > 0.05), thereby fulfilling both assumptions required for further analysis.

Table 2 shows the means and standard deviations for coping strategies (CSI), health perception (GHQ-28), perceived stress (PSS), self-esteem (RSES), and caregiving experience (ECI) among siblings of individuals with SMDs. Regarding coping strategies, a greater use of problem-focused maladaptive coping strategies (*M* = 20.00, *SD* = 6.91) was identified, particularly wishful thinking (*M* = 13.52, *SD* = 5.10), when compared to other coping strategies. This group was found to report a high overall negative perception of health *(M* = 9.17, *SD* = 8.02). This perception was characterized by higher mean scores on anxiety/anguish (*M* = 3.11, *SD* = 2.55), followed by somatic symptoms (*M* = 2.38, *SD* = 2.08), social dysfunction (*M* = 2.31, *SD* = 2.42), and depression (*M* = 1.35, *SD* = 2.06).

Furthermore, the group showed difficulties in self-esteem, with scores falling very close to the lower limit of the “normal” range (*M* = 25.32, *SD* = 2.19), as well as problems with perceived stress (*M* = 28.75, *SD* = 8.57) with negative stress (*M* = 16.38, *SD* = 4.97) being higher than positive stress (*M* = 12.37, *SD* = 4.33). Last, in terms of the caregiving experience, negative perceptions (*M* = 124.77, *SD* = 22.54) predominated over positive perceptions (*M* = 34.83, *SD* = 12.22), with the most prominent domains being difficult behaviors (*M* = 17.13, *SD* = 7.53), negative symptoms (*M* = 15.62, *SD* = 5.58), losses (*M* = 14.2, *SD* = 4.64), and problems with social and health services (*M* = 13.71, *SD* = 6.28).

To assess whether there were significant differences between the four coping strategies, repeated measures ANOVA was performed. Prior to this analysis, the sphericity assumption was checked. Mauchly’s test indicated that the assumption was violated (*p* = 0.002) and, therefore, the Greenhouse-Geisser correction was used to interpret the ANOVA results. The ANOVA showed that the difference in the mean scores of the coping strategies was statistically significant, *F_(_*_3, 177)_ = 5.657, *p* = 0.001, with a moderate effect size (η^2^ = 0.087). Bonferroni post hoc tests indicated that the main differences were between PFMC strategies and EFMC strategies (*p* = 0.002), where the former are used to a greater extent than the latter, and between PFAC strategies and EFMC strategies (*p* = 0.004), where the former are used to a greater extent than the latter (Table 3).

### 3.2. Correlational Analysis

Table 4 presents the correlations (Pearson’s *r*) between the different psychological variables assessed. Emotion-focused maladaptive coping (EFMC) showed the most extensive pattern of correlations, being positively associated with positive caregiving appraisal (*r* = 0.83, 95% CI [0.72, 0.89], *p* < 0.001), negative stress (*r* = 0.54, 95% CI [0.33, 0.70], *p* < 0.001), depression (*r* = 0.53, 95% CI [0.31, 0.69], *p* < 0.001), overall health (*r* = 0.49, 95% CI [0.27, 0.66], *p* < 0.001), social dysfunction (*r* = 0.47, 95% CI [0.25, 0.65], *p* < 0.001), anxiety/anguish (*r* = 0.42, 95% CI [0.19, 0.61], *p* < 0.001), positive stress (*r* = 0.36, 95% CI [0.12, 0.56], *p* < 0.01), somatic symptoms (*r* = 0.31, 95% CI [0.06, 0.52], *p* < 0.05), and negative caregiving appraisal (*r* = 0.24, 95% CI [−0.02, 0.46], *p* < 0.05). Conversely, a negative correlation was found with self-esteem (*r* = −0.39, 95% CI [−0.59, −0.15], *p* < 0.01).

Next, problem-focused maladaptive coping (PFMC), which also showed positive and significant correlations with the positive caregiving appraisal scales (*r* = 0.73, 95% CI [0.58, 0.83], *p* < 0.001), negative stress (*r* = 0.33, 95% CI [0.09, 0.54], *p* < 0.01), positive stress (*r* = 0.33, 95% CI [0.09, 0.54], *p* < 0.01), anxiety/anguish (*r* = 0.30, 95% CI [0.05, 0.52], *p* < 0.05), negative caregiving appraisal (*r* = 0.30, 95% CI [0.05, 0.52], *p* < 0.05), and overall health (*r* = 0.26, 95% CI [0.00, 0.48], *p* < 0.05).

In contrast, problem-focused adaptive coping (PFAC) showed a more limited pattern of correlations. PFAC was inversely associated with positive stress (*r* = −0.28, 95% CI [−0.50, −0.03], *p* < 0.05) and positive caregiving appraisal (*r* = −0.25, 95% CI [−0.47, −0.00], *p* < 0.05) and directly associated with negative caregiving appraisal (*r* = 0.26, 95% CI [0.00, 0.48], *p* < 0.05). Similarly, emotion-focused adaptive coping *(*EFAC) was only positively related to negative caregiving appraisal (*r* = 0.45, 95% CI [0.22, 0.63], *p* < 0.001).

We also evaluated the degree of relationship between the different coping strategies. We found that EFMC had a positive relationship with PFMC (*r* = 0.33, 95% CI [0.09, 0.54], *p* < 0.01) and a negative relationship with PFAC (*r* = −0.29, 95% CI [−0.50, −0.03], *p* < 0.01). Additionally, PFAC had a positive relationship with EFAC (*r* = 0.39, 95% CI [0.15, 0.58], *p* < 0.01).

### 3.3. Regression Analysis

Linear regression analyses were performed to identify which coping strategies (PFAC, EFAC, PFMC, EFMC) predicted overall health (and specific aspects: Somatic symptoms, anxiety/anguish, social dysfunction, depression), self-esteem, stress (positive and negative), and (positive and negative) caregiving appraisal (Table 5).

The regression model for somatic symptoms was not statistically significant, *F*(4, 55) = 2.228, *p* > 0.05. The four coping strategies explained a limited portion of the total variance in somatic symptoms (*R*^2^ = 0.139). Collinearity statistics (max VIF = 1.15, min Tolerance = 0.772) for all predictor variables were within acceptable limits, confirming the absence of multicollinearity issues in the model. In this case, despite individual coefficients appearing statistically significant, the overall model lacks significance, suggesting that the variables collectively do not adequately explain the outcome’s variability. Nonetheless, the observed trend suggests an association between EFMC strategies and somatic symptoms (*B* = 0.078, *SE* = 0.036, *t* = 2.196, *p* = 0.032, 95% CI [0.007, 0.150]).

The regression model for anxiety/anguish was statistically significant, *F*(4, 55) = 3.660, *p* ≤ 0.01, and explained 21.0% of the variance in this variable (*R*^2^ = 0.210, adjusted *R*^2^ = 0.153). Upon examining individual predictors among the four coping strategies, only EFMC was a significant positive predictor (*B* = 0.117, *SE* = 0.042, *t* = 2.794, *p* < 0.01, 95% CI [0.033, 0.201]). This indicates that, after controlling for other predictors, higher use of EFMC is significantly associated with an increase in anxiety/insomnia levels. Additionally, collinearity statistics (max VIF = 1.15, min Tolerance = 0.772) were acceptable for all predictors, ruling out multicollinearity issues.

The regression model for social dysfunction was statistically significant, *F*(4, 55) = 4.316, *p* < 0.01, explaining 23.9% of the total variance in this variable (*R*^2^ = 0.239, adjusted *R*^2^ = 0.184). Upon examining individual predictors, the only variable that demonstrated a unique and significant contribution to the model was EFMC (*B* = 0.129, *SE* = 0.039, *t* = 3.318, *p* < 0.01, 95% CI [0.051, 0.207]). This indicates that, independent of other factors, higher use of EFMC is strongly associated with an increase in social dysfunction levels. Collinearity assessment (max VIF = 1.296, min Tolerance = 0.772) confirmed the absence of multicollinearity issues among the predictors.

The regression model for depression was significant, *F*(4, 55) = 6.065, *p* < 0.001, explaining 30.6% of the variance in this variable (*R*^2^ = 0.306, adjusted *R*^2^ = 0.256). Upon examining individual predictors, EFMC made a unique, positive, and significant contribution to depression (*B* = 0.128, *SE* = 0.032, *p* < 0.001, 95% CI [0.065, 0.191]). This indicates that, holding other variables constant, higher use of this coping style is directly associated with higher levels of depression. Collinearity statistics (max VIF = 1.296, min Tolerance = 0.772) confirmed the absence of multicollinearity among the predictors.

The regression model for *global health* was statistically significant, *F*(4, 55) = 4.952, *p* < 0.01, explaining 26.5% of the variance in this variable (*R*^2^ = 0.265, adjusted *R*^2^ = 0.211). Upon examining individual predictors, EFMC made a unique, positive, and significant contribution to global health scores (*B* = 0.452, *SE* = 0.127, *t* = 3.565, *p* < 0.001, 95% CI [0.198, 0.707]). This indicates that, after controlling for other predictors, higher use of this coping style is directly associated with higher scores, reflecting a poorer perception of global health. Collinearity statistics (max VIF = 1.296, min Tolerance = 0.772) confirmed the absence of multicollinearity among the predictors.

The regression model for self-esteem was statistically significant, *F*(4, 55) = 3.364, *p* < 0.05, and explained 19.7% of the variance in this variable (*R*^2^ = 0.197, adjusted *R*^2^ = 0.138). Upon examining individual predictors among the four coping strategies, only EFMC was a significant negative predictor (*B* = −0.106, *SE* = 0.036, *t* = −2.937, *p* < 0.01, 95% CI [−0.179, −0.034]). This indicates that, independent of other factors, higher use of EFMC is significantly associated with a decrease in self-esteem levels. Additionally, collinearity statistics (max VIF = 1.296, min Tolerance = 0.772) were acceptable for all predictors, ruling out multicollinearity issues.

The regression model for positive stress was statistically significant, *F*(4, 55) = 4.058, *p* < 0.01, and explained 22.8% of the variance in this variable (*R*^2^ = 0.228, adjusted *R*^2^ = 0.172). Upon examining individual predictors, only PFAC was a significant negative predictor of positive stress (*B* = −0.148, *SE* = 0.074, *t* = −2.014, *p* < 0.05, 95% CI [−0.296, −0.001]). Holding other variables constant, higher PFAC use significantly correlates with lower positive stress, evidencing greater perceived control and coping capacity. Collinearity statistics (max VIF = 1.296, min Tolerance = 0.772) for all predictor variables were within acceptable limits, confirming the absence of multicollinearity in the model.

The regression model for negative stress was statistically significant, *F*(4, 55) = 7.204, *p* < 0.001, explaining 34.4% of the variance in this variable (*R*^2^ = 0.344, adjusted *R*^2^ = 0.296). Upon examining individual predictors, EFMC made a unique, positive, and significant contribution to negative stress (*B* = 0.277, *SE* = 0.074, *t* = 3.740, *p* < 0.001, 95% CI [0.129, 0.426]). This indicates that, after controlling for other predictors, higher use of EFMC is directly associated with higher levels of negative stress. Collinearity statistics (max VIF = 1.296, min Tolerance = 0.772) confirmed the absence of multicollinearity among the predictors.

The regression model for the positive caregiving appraisal was statistically significant, *F*(4, 55) = 146.327, *p* < 0.001, explaining 91.4% of the variance in this variable (*R*^2^ = 0.914, adjusted *R*^2^ = 0.908). Upon examining individual predictors, two made unique and significant contributions: PFMC (*B* = 0.066, *SE* = 0.005, *t* = 12.006, *p* < 0.001) and EFMC (*B* = 0.073, *SE* = 0.005, *t* = 15.122, *p* < 0.001, 95% CI [−0.010, 0.083]). Contrary to expectations, these results indicate that higher use of maladaptive coping strategies is associated with a greater perception of the positive aspects of caregiving. Strategies classified as adaptive were not significant in this model. The absence of multicollinearity was confirmed (max VIF = 1.296, min Tolerance = 0.772).

The regression model for the negative caregiving appraisal was statistically significant, *F*(4, 55) = 8.418, *p* < 0.001, explaining 38.0% of the variance in this variable (*R*^2^ = 0.380, adjusted *R*^2^ = 0.335). Two strategies showed a marginal unique contribution to the increase in negative perceptions: EFAC (*B* = 0.020, *SE* = 0.007, *t* = 1.826, *p* < 0.073, 95% CI [−0.006, 0.034]) and EFMC (*B* = 0.024, *SE* = 0.009, *t* = 2.741, *p* = 0.01, 95% CI [0.006, 0.042]). These results suggest that as caregivers rely more on emotion-focused coping strategies (both adaptive and maladaptive), their perception of caregiving burden increases. Problem-focused strategies were not significant. Collinearity statistics (max VIF = 1.296, min Tolerance = 0.772) confirmed the absence of multicollinearity.

A comparative analysis of the regression models reveals that EFMC emerged as the most consistent and powerful predictor across nearly all analyzed dimensions. Specifically, EFMC demonstrated a significant positive association with depression, poor perceived health, negative stress, social dysfunction, and anxiety. Conversely, it was the only significant negative predictor of self-esteem. Remarkably, even in the model for positive aspects of caregiving, EFMC maintained a strong predictive role, albeit in an unexpected direction. In contrast, problem-focused and emotional-focused strategies failed to reach statistical significance in most models. These findings suggest that the reliance on emotional-focused strategies –regardless of the specific psychological outcome– constitutes the primary factor explaining the variance in this sample.

The reliability and validity of the regression model for predicting somatic symptoms, anxiety/anguish, social dysfunction, depression, self-esteem, stress (positive and negative), and (positive and negative) caregiving appraisal were assessed by examining the distribution of the residuals and identifying influential observations (see Supplementary Material).

The assumption of homoscedasticity (constant error variance) was evaluated using a scatter plot of the Residuals versus Predicted Values. As shown in Figures S1, S3, S5, S7, S9, S11, S13, S15, S17 and S19, the points representing the residuals appear to be randomly scattered around the zero line, without displaying any discernible pattern (e.g., a funnelling effect, U-shaped distributions). This random dispersion suggests that the assumption of homoscedasticity is reasonably met, and there is no clear evidence of non-linearity.

The assumption of normality of the errors was visually inspected using a Q-Q Plot of the Standardized Residuals. As shown in Figures S2, S4, S6, S8, S10, S12, S14, S16, S18 and S20, the majority of the data points closely follow the diagonal reference line, indicating that the distribution of the residuals approximates a normal distribution. Sometimes, a slight deviation from the line was noted at the extreme tails, but this was not considered severe enough to significantly violate the assumption for the purposes of this analysis (e.g., Figure S2).

To evaluate the influence of individual cases on the stability of the regression coefficients, Cook’s Distance was calculated (Tables S1–S10—Supplementary Material). For our sample size (*N* = 60), a potentially problematic influence will be when Cook’s Distance exceeds the value of 4/*N* (≈ 0.067). For example, regarding the regression model for depression, the analysis of residuals confirmed the fulfillment of linear regression assumptions. The Residuals vs. Predicted plot (Figure S7) showed no evidence of heteroscedasticity, and the Q-Q plot (Figure S8) indicated a normal distribution of errors. While a few cases (24, 33, 34, 39, and 41) slightly exceeded the 4/*N* threshold (max value = 0.124), they remained well below the commonly accepted critical value of 1.0, indicating that no single observation exerted an undue or distorting influence on the model parameters; therefore, no unduly influential observations were identified that would necessitate removal or suggest a significant instability in the model’s coefficients.

## 4. Discussion

The aim of this study was to identify the coping strategies utilized by siblings of individuals with SMDs and to analyze their relationship with perceived health, perceived stress, self-esteem, and caregiving experience. To the best of our knowledge, this study is pioneering in the field of mental health research concerning this specific population, thus opening the door for further analysis of the findings. The results obtained have important implications for a better understanding of the experience of this population group. Furthermore, they are relevant to the organization of mental health services and the design of mental health care policies for the families of individuals affected by an SMD.

A first important finding was that siblings utilize problem-focused strategies -both adaptive and maladaptive- to a greater extent than emotion-focused maladaptive strategies. This suggests that siblings tend to focus their coping resources on attempting to solve problems, rather than on managing the emotional aspects involved in difficult or complex situations, such as caring for a sibling with an SMD [1,19,25,43]. Since no significant differences were found between the use of maladaptive and adaptive strategies (both of which are problem-focused), we can assume that siblings make significant active efforts, both cognitively and behaviorally, to face the challenges of these difficult situations. Furthermore, the lack of a relationship between the two types of strategies suggests that these efforts are highly diverse and encompass various ways of coping. This can be understood by considering coping as a dynamic and interactive process between the individual and the environment, where the strategies employed depend on the person’s appraisal of the demands and the resources perceived to meet them [15]. Our data appear to contrast with previous studies that emphasize the use of maladaptive problem-focused strategies (e.g., avoidance, denial, or resignation) by relatives of people with SMDs, particularly when they perceive little control over the patient’s disorder or behavior [18,19,25,27,44,45,46,47]. Concerning these maladaptive problem-focused strategies, our study also identified “wishful thinking,” which reflects the desire for reality to be different or the attempt to avoid the situation and its stressful nature [31,32]. In contrast to our findings, there is little mention in the literature regarding the use of strategies such as cognitive restructuring or problem solving. These strategies are considered adaptive because they aim to assign a different meaning to the situation or to find a way to solve what is possible. In summary, our study indicates that coping modes focused on the problem are more prevalent than emotion-focused ones, although there is an undifferentiated use of adaptive and maladaptive efforts within this coping style.

To assess the implications that the use of the four types of coping strategies may have on aspects such as perceived health, perceived stress, self-esteem, and the appraisal of the siblings’ caregiving experience, we analyzed the relationships between these psychological variables. The results seem to indicate that, in general, maladaptive coping -both emotion-focused (EFMC) and problem-focused (PFMC)- are related to greater psychological difficulties. These strategies are associated with emotionally ineffective coping, characterized by high affective load, difficulties in the caregiver role, and self-esteem. These findings align with studies that highlight that the predominant use of maladaptive strategies tends to amplify emotional stress and increase the risk of caregiver overload and mental health deterioration [18,22].

Of particular note is the crucial role played by EFMC strategies. Paradoxically, while their use is significantly related to high levels of positive caregiving appraisal, it is also associated with higher levels of depression, poorer perceived health, negative stress, social dysfunction, and anxiety, as well as lower self-esteem. This coping pattern, characterized by reactive emotional responses, such as rumination, avoidance, and self-criticism, increases psychological vulnerability and impairs both emotional balance and self-perception. In this context, the sustained use of EFMC is not only ineffective in reducing distress but also contributes to its chronicity, thereby reinforcing a persistent cycle of emotional stress in family caregivers [48,49]. These results are consistent with previous research highlighting the detrimental role of coping with prolonged stress in family caregiving contexts [19,21,50]. From a family policy and public health perspective, the results underscore the need for institutional recognition of the non-parental caregiving role assumed by siblings of individuals with SMDs [18,27,51,52].

Notably, the results show that EFMC, in conjunction with PFMC, predicts the variance in positive caregiving appraisal. This suggests that utilizing both types of strategies may increase role satisfaction and strengthen the relationship with siblings diagnosed with SMDs. The association between a positive caregiving appraisal and the use of maladaptive emotional strategies can be understood through the ambivalence inherent in the caregiver role [49]. In this sense, emotional strategies could increase affective involvement and reinforce feelings of responsibility, closeness, or sense of duty, thereby promoting a positive assessment of the role. However, this very process simultaneously entails high emotional costs [27]. In summary, our study indicates that EFMC are the strongest and most consistent predictor of psychological distress in siblings of people with SMDs, demonstrating how maladaptive emotional responses sustain a persistent cycle of mental health problems and low self-esteem [14,50], and contrary to expectations– a positive caregiving appraisal.

On the other hand, adaptive strategies—both PFAC and EFAC—appear to be less related to psychological distress. In fact, PFAC was associated with a reduction in positive stress factor; this could indicate that when siblings attempt to navigate the complexities of SMDs, their appraisal focuses on identifying the caregiving challenges and obstacles, while perceiving that their efforts enable them, to some extent, to maintain control and manage situations skillfully. Regarding EFAC, these appear to be primarily linked to a more critical or realistic appraisal of the negative impact of caregiving, as they increase the perception of caregiver burden by highlighting the associated losses, sacrifices, and difficulties, particularly in chronic stress contexts where external conditions remain unchanged. This finding is especially relevant considering that, together with PFMC, they constitute a key factor in the negative appraisal of the caregiving experience. These results suggest that an emotion-focused coping style (even when adaptive), when combined with inadequate PFMC strategies in high-emotional-load contexts, does not necessarily improve the caregiver’s experience. On the contrary, it contributes to intensifying the negative assessment of the situation [53].

This overview of how different coping strategies are related to certain psychological problems has important clinical implications. First, given the scarcity of interventions specifically targeting siblings of people with SMDs [24], it seems appropriate that family interventions should have among their central goal the development or reinforcement of coping skills. It is a priority that mental health policies promote the development of family intervention programs designed to better manage distress, foster adaptive coping skills, and reduce the inequalities associated with this often invisible caregiving experience, especially among siblings [18,27,51,52]. An example of this “invisibility” within mental health services is the high percentage (86.7%) of participants in this study who were unaware of their sibling’s specific diagnostic label. The implementation of group psychological programs within the public mental health network of Granada (Spain) is particularly relevant, given the framework of the community model and the existing synergies between the healthcare system, the university, and family associations.

At the intervention level, it should focus especially on reducing the presence of EFMC, as these are the ones most involved in the psychological maladjustments of siblings. Therefore, it would be advisable to enhance skills for emotional regulation, communication, and problem solving, complemented by strategies to reduce stress and anxiety, and improve social recovery [18,19,27,43]. Second, these psychological interventions serve as preventive (rather than solely rehabilitative) measures for such psychological problems. These measures could be implemented with family members, particularly siblings, from the early stages of SMD diagnosis [21], and this should be a central component of public healthcare policy. Implementing early detection and intervention in mental health services could effectively prevent the onset of future complications among these patients’ siblings. Furthermore, such interventions could promote a more balanced caregiving experience by fostering adaptive strategies and reducing the emotional burden associated with the caregiver role. In line with the community-based approach promoted by the Spanish Mental Health Strategy 2022–2026 [51], these findings support the need to broaden the focus of intervention beyond the patient. This broadening includes siblings as a target population throughout the care process: From assessment and prevention to psychological intervention in mental health units.

This study has several limitations that should be considered when interpreting the results. The small sample size (*N* = 60) limits the generalizability of the findings, may affect the stability of the regression models, and increases the risk of Type I errors, necessitating a cautious interpretation of the results. Furthermore, the limited number of participants precluded the performance of gender-stratified analyses. The recruitment procedure, which required prior patient consent, may have introduced selection bias by favoring families with higher levels of functionality and collaboration, thereby compromising the representativeness of the sample. Other relevant limitations include the absence of detailed clinical information regarding patients (e.g., clinical diagnosis, current severity level), the exclusive use of self-report measures – which may inflate the observed associations due to common-method variance–, the cross-sectional design, which prevents establishing causal relationships, and the lack of a comparison group, which makes it difficult to assess the specificity of the findings in relation to other caregivers.

Based on the limitations discussed above, we consider it pertinent that future research expand the sample size and diversify the contexts from which participants are recruited. It would also be advisable to incorporate detailed clinical information on patients, expand the use of data collection methods (e.g., use in-depth interviews, focus groups), and include longitudinal designs, and explore mediating and moderating variables (e.g., resilience, grief, social support, and objective care burden). Finally, it would be valuable to design and evaluate psychological interventions specifically aimed at siblings of individuals with SMDs, with the goal of strengthening their personal resources and promoting more adaptive coping with their sibling’s disorder.

## 5. Conclusions

This study represents a pioneering contribution to research on the mental health of siblings of patients with SMDs. The results indicate that although siblings extensively use problem-focused coping strategies (both adaptive and maladaptive), it is the emotion-focused maladaptive coping (EFMC) strategies that are strongly associated with detrimental outcomes for this population. Specifically, our findings suggest that reliance on EFMC acts as a risk factor that undermines psychological well-being, self-esteem, and the siblings’ overall caregiving experience. These results highlight the importance of promoting psychological interventions focused on emotional regulation that strengthen personal resources and social support. Finally, the relevance of developing targeted public mental health policies must be noted. Given that early-stage psychological interventions for populations such as this could improve coping mechanisms in the complex situation of having a sibling with SMDs, they would consequently mitigate the personal emotional impact and the broader burden on the healthcare system.

## Figures and Tables

**Table 1:** Sociodemographic and contextual characteristics of participants (*N* = 60).

Characteristics	*n*	%
Gender		
Female	37	61.7
Men	23	38.3
With partner		
Yes	26	43.3
No	34	56.6
Level of education		
Primary	8	13.3
Secondary	26	43.3
University	26	43.3
Employment status		
Paid	32	53.3
Unemployed/Out of work	9	14.9
Housewife	3	5.0
Student	16	26.6
Knowledge of the patient’s diagnostic label		
Yes	8	13.3
No	52	86.7
Perceived disorder duration (years)		
<5	22	36.6
5–10	21	35.0
>10	17	28.3
Primary caregiver status		
Yes	7	11
No	53	89
Co-residence with patient		
Yes	37	61
No	23	39

**Table 2:** Means and standard deviations of the psychological variables assessed in the siblings of patients with severe mental disorder.

Variable (Instrument)/Subscales	*M*	*SD*	Min–Max
Coping strategies (CSI)			
Problem-focused adaptive coping	19.72	7.94	0–40
Problem solving	11.02	5.12	0–20
Cognitive restructuring	8.70	4.07	0–20
Emotion-focused adaptive coping	18.57	9.05	0–40
Social support	9.25	5.67	0–20
Express emotion	9.32	4.82	0–20
Problem-focused maladaptive coping	20.0	6.91	0–40
Problem avoidance	6.48	4.46	0–20
Wishful thinking	13.52	5.10	0–20
Emotion-focused maladaptive coping	14.83	8.03	0–40
Self-criticism	7.77	5.41	0–20
Social withdrawal	7.17	4.75	0–20
Perceived health (GHQ-28)			
Somatic symptoms	2.38	2.08	0–7
Anxiety/anguish	3.11	2.55	0–7
Social dysfunction	2.31	2.42	0–7
Depression	1.35	2.06	0–7
Overall	9.17	8.02	0–28
Self-esteem (RSES)	25.32	2.19	10–40
Perceived stress (PSS)			
Negative stress	16.38	4.97	0–28
Positive stress	12.37	4.33	0–28
Overall	28.75	8.57	0–56
Caregiving experience (ECI)			
Negative appraisal	124.77	22.54	0–208
Difficult behaviors	17.13	7.54	0–32
Negative symptoms	15.62	5.58	0–24
Stigma	7.47	4.43	0–20
Problems with social and health services	13.72	6.23	0–32
Consequences for the family	11.70	5.49	0–28
Need for backing and support	11.85	4.31	0–24
Dependency	10.52	3.48	0–20
Loss	14.20	4.65	0–28
Positive appraisal	34.83	12.22	0–56
Satisfactory personal experiences	17.27	5.37	0–32
Positive aspects of the relationship	12.25	4.23	0–24

Note: *N* = 60. CSI = Coping Strategies Inventory; GHQ-28 = General Health Questionnaire; RSES = Rosenberg Self-Esteem Scale; PSS = Perceived Stress Scale; ECI = Caregiving Inventory.

**Table 3:** Post hoc comparisons of coping strategies.

Variables		Mean Difference	SE	*t*	*p*_Bonf_
PFAC	EFAC	1.150	1.415	0.813	1.000
	PFMC	−0.283	1.415	−0.200	1.000
	EFMC	4.883	1.415	3.452	0.004 **
EFAC	PFMC	−1.433	1.415	−1.013	1.000
	EFMC	3.733	1.415	2.639	0.054
PFMC	EFMC	5.167	1.415	3.652	0.002

Note: Bonf = Bonferroni; PFAC = Problem-focused adaptive coping; EFAC= Emotion-focused adaptive coping; PFMC = Problem-focused maladaptive coping; EFMC = Emotion-focused maladaptive coping. ** *p* < 0.01.

**Table 4:** Correlational analyses.

Variables	1	2	3	4	5	6	7	8	9	10	11	12	13
1. Somatic symptoms	—												
2. Anxiety/anguish	0.69 ***	—											
3. Social dysfunction	0.64 ***	0.74 ***	—										
4. Depression	0.69 ***	0.68 ***	0.78 ***	—									
5. Global health	0.84 ***	0.89 ***	0.90 ***	0.88 ***	—								
6. Self-esteem	−0.31 *	−0.28 *	−0.31 *	−0.46 ***	−0.38 **	—							
7. Negative stress	0.48 ***	0.52 ***	0.58 ***	0.59 ***	0.62 ***	−0.42 ***	—						
8. Positive stress	0.37 **	0.41 **	0.45 ***	0.43 ***	0.48 ***	−0.29 *	0.70 ***	—					
9. Negative appraisal	0.39 ***	0.37 ***	0.44 ***	0.43 ***	0.46 ***	−0.31 *	0.64 ***	0.54 ***					
10. Positive appraisal	0.30 *	0.49 ***	0.43 ***	0.44 ***	0.47 ***	−0.37 *	0.52 ***	0.41 ***	0.36 **				
11. PFAC	0.03	−0.10	−0.21	−0.20	−0.14	0.13	−0.25	−0.28 *	0.25 *	−0.25 *	—		
12. EFAC	0.19	0.06	0.03	0.12	0.11	−0.16	0.09	0.10	0.45 ***	0.04	0.39 **	—	
13. PFMC	0.19	0.30 *	0.21	0.19	0.26 *	−0.10	0.33 **	0.33 **	0.39 *	0.73 ***	−0.12	0.11	—
14. EFMC	0.31 *	0.42 ***	0.47 ***	0.53 ***	0.49 ***	−0.39 **	0.54 ***	0.36 **	0.24 *	0.83 ***	−0.29 *	−0.04	0.33 **

Note: Negative appraisal = Negative caregiving appraisal; Positive appraisal = Positive caregiving appraisal; PFAC = Problem-focused adaptive coping; EFAC = Emotion-focused adaptive coping; PFMC = Problem-focused maladaptive coping; EFMC = Emotion-focused maladaptive coping. * *p* < 0.05; ** *p* < 0.01; *** *p* < 0.001.

**Table 5:** Regression models.

CV (Instrument)	PV	B	*SE*	β	*t*	*p*	95% CI for B		Tolerance	VIF	*F*	*R* ^2^	Adj. *R*^2^
							Lower	Upper					
Somatic symptoms (GHQ)										*F*_(4,55)_ = 2.228		0.139	0.077
	PFAC	0.015	0.037	0.056	0.391	0.697	−0.060	0.090	0.772	1.296			
	EFAC	0.039	0.032	0.171	1.240	0.220	−0.024	0.103	0.826	1.211			
	PFMC	0.022	0.040	0.073	0.547	0.587	−0.059	0.103	0.868	1.153			
	EFMC	0.078	0.036	0.302	2.196	**0.032**	0.007	0.150	0.828	1.208			
Anxiety/anguish (GHQ)										*F*_(4,55)_ = 3.660 **		0.210	0.153
	PFAC	0.004	0.044	0.012	0.084	0.933	−0.084	0.092	0.772	1.296			
	EFAC	0.013	0.037	0.047	0.356	0.723	−0.061	0.088	0.826	1.211			
	PFMC	0.065	0.047	0.176	1.372	0.176	−0.030	0.160	0.868	1.153			
	EFMC	0.117	0.042	0.368	2.794	**0.007**	0.033	0.201	0.828	1.208			
Social dysfunction (GHQ)										*F*_(4,55)_ = 4.316 **		0.239	0.184
	PFAC	−0.034	0.041	−0.111	−0.821	0.409	−0.116	0.048	0.772	1.296			
	EFAC	0.021	0.035	0.080	0.616	0.540	−0.048	0.091	0.826	1.211			
	PFMC	0.017	0.044	0.048	0.378	0.707	−0.072	0.105	0.868	1.153			
	EFMC	0.129	0.039	0.429	3.318	**<0.002**	0.051	0.207	0.828	1.208			
Depression (GHQ)										*F*_(4.55)_ = 6.065 ***		0.306	0.256
	PFAC	−0.033	0.033	−0.127	−0.991	0.326	−0.099	0.034	0.772	1.296			
	EFAC	0.042	0.028	0.185	1.500	0.139	−0.014	0.098	0.826	1.211			
	PFMC	−0.005	0.036	−0.015	−0.126	0.901	−0.076	0.067	0.868	1.153			
	EFMC	0.128	0.032	0.500	4.049	**<0.001**	0.065	0.191	0.828	1.208			
Overall health (GHQ)										*F*_(4.55)_ = 4.952 **		0.265	0.211
	PFAC	−0.048	0.133	−0.048	−0.364	0.717	−0.315	0.218	0.772	1.296			
	EFAC	0.116	0.113	0.131	1.029	0.308	−0.110	0.342	0.826	1.211			
	PFMC	0.099	0.144	0.086	0.691	0.493	−0.189	0.388	0.868	1.153			
	EFMC	0.452	0.127	0.453	3.565	**<0.001**	0.198	0.707	0.828	1.208			
Self-esteem (RSES)										*F*_(4.55)_ = 3.364 *		0.197	0.138
	PFAC	0.030	0.038	0.109	0.795	0.430	−0.046	0.106	0.772	1.296			
	EFAC	−0.055	0.032	−0.225	−1.693	0.096	−0.119	0.010	0.826	1.211			
	PFMC	0.021	0.041	0.065	0.501	0.618	−0.062	0.103	0.868	1.153			
	EFMC	−0.106	0.036	−0.390	−2.937	**<0.005**	−0.179	−0.034	0.828	1.208			
Positive stress (PSS)										*F*_(4,55)_ = 4.058 **		0.228	0.172
	PFAC	−0.148	0.074	−0.272	−2.014	**0.049**	−0.296	0.000	0.772	1.296			
	EFAC	0.090	0.062	0.188	1.439	0.156	−0.035	0.215	0.826	1.211			
	PFMC	0.130	0.080	0.208	1.636	0.108	−0.029	0.290	0.868	1.153			
	EFMC	0.105	0.070	0.194	1.493	0.141	−0.036	0.245	0.828	1.208			
Negative stress (PSS)										*F*_(4,55)_ = 7.204 ***		0.344	0.296
	PFAC	−0.100	0.078	−0.160	−1.290	0.202	−0.256	0.056	0.772	1.296			
	EFAC	0.081	0.066	0.147	1.224	0.226	−0.051	0.213	0.826	1.211			
	PFMC	0.106	0.084	0.148	1.259	0.213	−0.063	0.245	0.868	1.153			
	EFMC	0.277	0.074	0.449	3.740	**<0.001**	0.129	0.426	0.828	1.208			
Positive care appraisal (ECI)										*F*_(4,55)_ = 146.327 ***		0.914	0.908
	PFAC	0.000	0.005	−0.001	−0.014	0.989	−0.010	0.010	0.772	1.296			
	EFAC	0.000	0.004	0.004	0.103	0.919	−0.008	0.009	0.826	1.211			
	PFMC	0.066	0.005	0.509	12.006	**<0.001**	0.055	0.868	0.868	1.153			
	EFMC	0.073	0.005	0.657	15.122	**<0.001**	0.063	0.828	0.828	1.208			
Negative care appraisal (ECI)										*F*_(4.55)_ = 8.418 ***		0.380	0.335
	PFAC	0.015	0.008	0.221	1.826	0.073	−0.001	0.031	0.772	1.296			
	EFAC	0.020	0.007	0.339	2.902	**0.005**	0.006	0.034	0.826	1.211			
	PFMC	0.024	0.009	0.312	2.741	**0.008**	0.006	0.042	0.868	1.153			
	EFMC	0.014	0.008	0.206	1.764	0.083	−0.002	0.029	0.828	1.208			

Note: CV = Criterion variables; PV = Predictor variables; Adj. *R*^2^ = Adjusted *R*^2^; GHQ = General Health Questionnaire; PFAC = Problem-focused adaptive coping; EFAC = Emotion-focused adaptive coping; PFMC = Problem-focused maladaptive coping; EFMC = Emotion-focused maladaptive coping; RSES = Rosenberg Self-Esteem Scale; PSS = Perceived Stress Scale; CSI = Coping Strategies Inventory; ECI = Caregiving Inventory. Significant values are in bold. * *p* < 0.05; ** *p* < 0.01; *** *p* < 0.001.

## Data Availability

The data presented in this study are available upon request to the corresponding author because they contain information that could compromise the confidentiality of the research subjects (ethical and privacy restrictions of the participants).

## Supplementary Materials

The following supporting information can be downloaded at: https://www.mdpi.com/article/10.3390/healthcare14030388/s1, **Somatic symptoms (GHQ)** Figure S1. Residuals vs. Predicted; Figure S2. Q-Q Plot Standardized Residuals; Table S1. Casewise Diagnostics; **Anxiety (GHQ)** Figure S3. Residuals vs. Predicted; Figure S4. Q-Q Plot Standardized Residuals; Table S2. Casewise Diagnostics; **Social dysfunction (GHQ)** Figure S5. Residuals vs. Predicted; Figure S6. Q-Q Plot Standardized Residuals; Table S3. Casewise Diagnostics; **Depression (GHQ)** Figure S7. Residuals vs. Predicted; Figure S8. Q-Q Plot Standardized Residuals; Table S4. Casewise Diagnostics; **Overall health (GHQ)** Figure S9. Residuals vs. Predicted; Figure S10. Q-Q Plot Standardized Residuals; Table S5. Casewise Diagnostics; **Self-esteem (RSES)** Figure S11. Residuals vs. Predicted; Figure S12. Q-Q Plot Standardized Residuals; Table S6. Casewise Diagnostics; **Positive stress (PSS)** Figure S13. Residuals vs. Predicted; Figure S14. Q-Q Plot Standardized Residuals; Table S7. Casewise Diagnostics; **Negative stress (PSS)** Figure S15. Residuals vs. Predicted; Figure S16. Q-Q Plot Standardized Residuals; Table S8. Casewise Diagnostics; **Positive care appreciation (ECI)** Figure S17. Residuals vs. Predicted; Figure S18. Q-Q Plot Standardized Residuals; Table S9. Casewise Diagnostics; **Negative care appreciation (ECI)** Figure S19. Residuals vs. Predicted; Figure S20. Q-Q Plot Standardized Residuals; Table S10. Casewise Diagnostics.

## Abbreviations

The following abbreviations are used in this manuscript:

CSI	Coping Strategies Inventory
ECI	Caregiving Inventory
GHQ-28	General Health Questionnaire-28
EFAC	Emotion-focused adaptive coping
EFMC	Emotion-focused maladaptive coping
PFMC	Problem-focused maladaptive coping
PFAC	Problem-focused adaptive coping
PSS	Perceived Stress Scale
RSES	Rosenberg Self-Esteem Scale
SMD	Severe mental disorder
