# Supplementary material for: Coping and Caregiving Experiences Among Siblings of Individuals with Severe Mental Disorders
Source: Healthcare (Basel). 2026 Feb 3;14(3):388. doi: 10.3390/healthcare14030388 (PMC12897001; doi:10.3390/healthcare14030388)
Supplement: Supplementary file 1 [file healthcare-14-00388-s001.zip › healthcare-4031818-supplementary.pdf]

Somatic symptoms (GHQ)

Figure S1. Residuals vs. Predicted

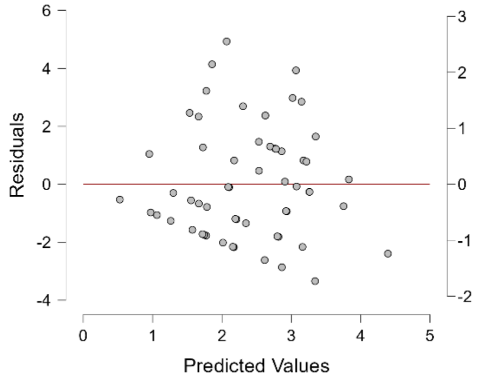

Figure S2. Q-Q Plot Standardized Residuals

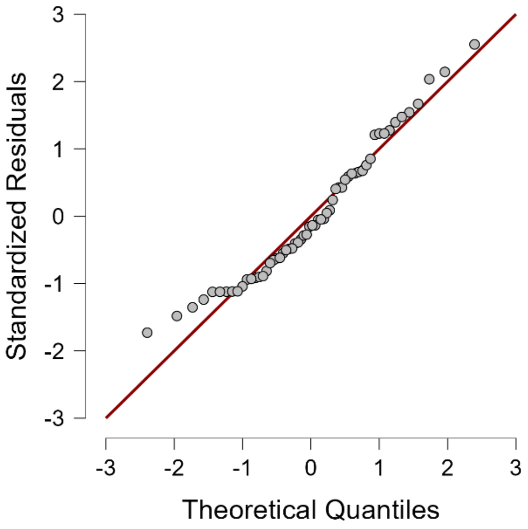

Table S1. Casewise Diagnostics

Case Number	Std. Residual	Síntomas somáticos	Predicted Value	Residual	Cook's Distance
1	0.626	4.000	2.768	1.232	0.003
2	-0.936	0.000	1.773	-1.773	0.021
3	0.431	3.000	2.177	0.823	0.004
4	-0.810	0.000	1.574	-1.574	0.008
5	0.653	3.000	1.727	1.273	0.004
6	-0.399	1.000	1.783	-0.783	0.001
7	1.374	5.000	2.304	2.696	0.016
8	-0.584	0.000	1.063	-1.063	0.014
9	-1.141	0.000	2.173	-2.173	0.027
10	-1.722	0.000	3.344	-3.344	0.037
11	2.105	6.000	1.858	4.142	0.031
12	0.088	4.000	3.831	0.169	0.000
13	-0.343	1.000	1.667	-0.667	0.001
14	-0.992	0.000	1.745	-1.745	0.058
15	1.210	4.000	1.664	2.336	0.022
16	-1.102	0.000	2.168	-2.168	0.009
17	-1.317	2.000	4.394	-2.394	0.074
18	-0.950	1.000	2.817	-1.817	0.017
19	-1.121	0.000	2.015	-2.015	0.060
20	0.591	4.000	2.862	1.138	0.006
21	-1.100	1.000	3.163	-2.163	0.009
22	-0.612	1.000	2.210	-1.210	0.002
23	-1.350	0.000	2.617	-2.617	0.024
24	1.574	6.000	3.020	2.980	0.059
25	0.427	4.000	3.176	0.824	0.003

Table S1. Casewise Diagnostics

Case Number	Std. Residual	Síntomas somáticos	Predicted Value	Residual	Cook's Distance
26	0.240	3.000	2.535	0.465	0.001
27	0.412	4.000	3.219	0.781	0.004
28	-0.039	3.000	3.076	-0.076	0.000
29	-0.053	2.000	2.105	-0.105	0.000
30	-1.122	0.000	2.171	-2.171	0.018
31	-0.134	3.000	3.260	-0.260	0.000
32	-1.100	0.000	2.157	-2.157	0.010
33	1.496	6.000	3.149	2.851	0.046
34	0.048	3.000	2.909	0.091	0.000
35	-0.929	1.000	2.799	-1.799	0.012
36	-0.392	3.000	3.755	-0.755	0.002
37	0.706	4.000	2.695	1.305	0.017
38	-0.670	0.000	1.263	-1.263	0.012
39	-0.498	2.000	2.935	-0.935	0.007
40	-0.155	1.000	1.298	-0.298	0.000
41	-0.512	2.000	2.925	-0.925	0.012
42	-0.051	2.000	2.092	-0.092	0.000
43	0.631	4.000	2.780	1.220	0.006
44	-0.681	1.000	2.347	-1.347	0.002
45	-0.284	1.000	1.555	-0.555	0.001
46	1.256	4.000	1.535	2.465	0.013
47	0.749	4.000	2.533	1.467	0.005
48	-0.881	0.000	1.721	-1.721	0.008
49	0.559	2.000	0.953	1.047	0.009
50	-0.519	0.000	0.973	-0.973	0.008
51	1.711	5.000	1.774	3.226	0.075
52	-0.135	3.000	3.264	-0.264	0.000
53	1.224	5.000	2.626	2.374	0.020
54	2.036	7.000	3.068	3.932	0.062
55	-1.479	0.000	2.865	-2.865	0.030
56	0.853	5.000	3.352	1.648	0.011
57	1.224	5.000	2.626	2.374	0.020
58	2.560	7.000	2.068	4.932	0.105
59	-0.288	0.000	0.527	-0.527	0.003
60	-0.618	1.000	2.194	-1.194	0.006

Anxiety (GHQ)

Figure S3. Residuals vs. Predicted

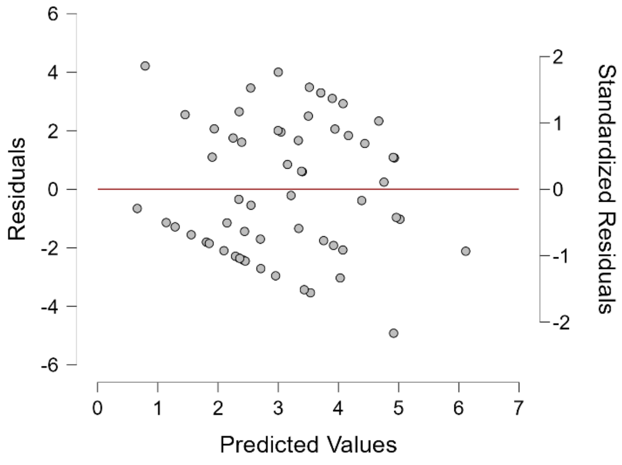

Figure S4. Q-Q Plot Standardized Residuals

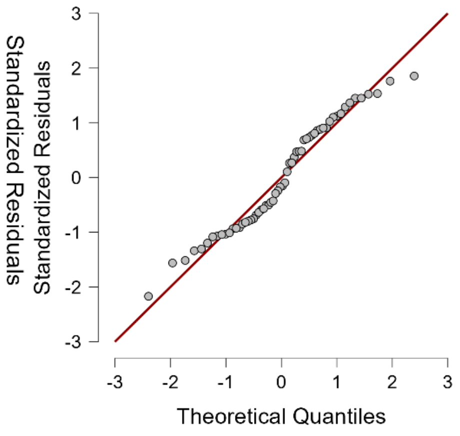

Table S2. Casewise Diagnostics

Case Number	Std. Residual	angustia/ansiedad	Predicted Value	Residual	Cook's Distance
1	1.508	7.000	3.517	3.483	0.016
2	-0.603	2.000	3.340	-1.340	0.009
3	-0.154	2.000	2.345	-0.345	0.000
4	-0.681	0.000	1.554	-1.554	0.006
5	-1.057	0.000	2.419	-2.419	0.012
6	0.896	4.000	1.937	2.063	0.006
7	0.367	4.000	3.154	0.846	0.001
8	-1.148	0.000	2.453	-2.453	0.055
9	-1.049	0.000	2.343	-2.343	0.023
10	-2.159	0.000	4.918	-4.918	0.059
11	1.498	6.000	2.543	3.457	0.016
12	0.108	5.000	4.758	0.242	0.000
13	0.765	4.000	2.251	1.749	0.007
14	-0.557	1.000	2.149	-1.149	0.018
15	1.124	4.000	1.453	2.547	0.019
16	-0.625	1.000	2.441	-1.441	0.003
17	-0.992	4.000	6.116	-2.116	0.042
18	-0.095	3.000	3.213	-0.213	0.000
19	-0.540	0.000	1.139	-1.139	0.014
20	-0.171	4.000	4.387	-0.387	0.000
21	-0.899	2.000	4.075	-2.075	0.006
22	0.842	5.000	3.046	1.954	0.004
23	-1.332	1.000	4.031	-3.031	0.023
24	1.125	6.000	3.501	2.499	0.030
25	0.473	6.000	4.931	1.069	0.004
26	0.882	5.000	2.997	2.003	0.011
27	0.490	6.000	4.912	1.088	0.006
28	-0.847	2.000	3.919	-1.919	0.011
29	-0.739	1.000	2.704	-1.704	0.004

Table S2. Casewise Diagnostics

Case Number	Std. Residual	angustia/ansiedad	Predicted Value	Residual	Cook's Distance
30	-1.009	0.000	2.290	-2.290	0.015
31	-0.772	2.000	3.755	-1.755	0.008
32	0.724	5.000	3.335	1.665	0.004
33	0.920	6.000	3.943	2.057	0.017
34	1.038	7.000	4.671	2.329	0.021
35	0.264	4.000	3.401	0.599	0.001
36	-0.452	4.000	5.023	-1.023	0.003
37	-0.252	2.000	2.547	-0.547	0.002
38	-0.817	0.000	1.806	-1.806	0.017
39	-0.437	4.000	4.963	-0.963	0.005
40	0.484	3.000	1.904	1.096	0.004
41	-1.668	0.000	3.537	-3.537	0.127
42	1.267	5.000	2.352	2.648	0.085
43	0.688	6.000	4.439	1.561	0.007
44	0.264	4.000	3.387	0.613	0.000
45	-0.810	0.000	1.853	-1.853	0.007
46	-0.911	0.000	2.098	-2.098	0.007
47	-1.286	0.000	2.957	-2.957	0.014
48	-1.183	0.000	2.711	-2.711	0.014
49	1.917	5.000	0.789	4.211	0.106
50	-0.585	0.000	1.286	-1.286	0.010
51	0.726	4.000	2.394	1.606	0.013
52	0.798	6.000	4.166	1.834	0.006
53	1.447	7.000	3.708	3.292	0.028
54	1.290	7.000	4.077	2.923	0.025
55	-1.510	0.000	3.432	-3.432	0.031
56	1.369	7.000	3.899	3.101	0.028
57	1.447	7.000	3.708	3.292	0.028
58	1.769	7.000	3.001	3.999	0.050
59	-0.306	0.000	0.658	-0.658	0.004
60	-1.041	0.000	2.361	-2.361	0.016

Social dysfunction (GHQ)

Figure S5. Residuals vs. Predicted

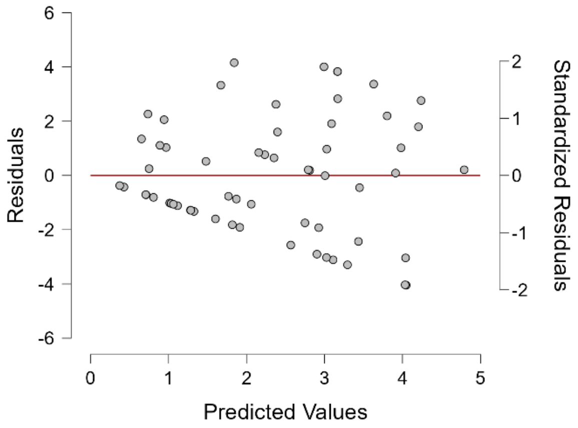

Figure S6. Q-Q Plot Standardized Residuals

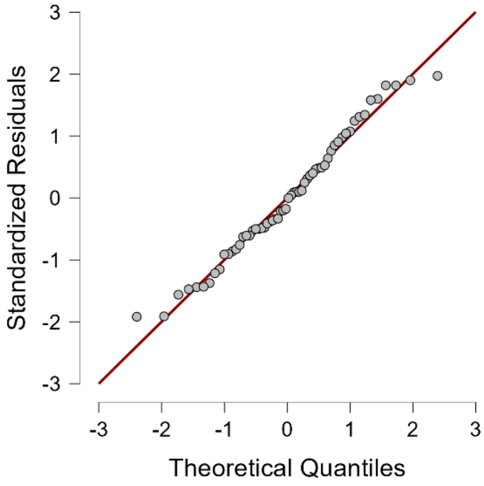

Table S3. Casewise Diagnostics

Case Number	Std. Residual	Disfunción social	Predicted Value	Residual	Cook's Distance
1	1.221	5.000	2.378	2.622	0.010
2	0.775	4.000	2.397	1.603	0.014
3	0.988	3.000	0.943	2.057	0.020
4	-0.478	0.000	1.015	-1.015	0.003
5	-0.498	1.000	2.061	-1.061	0.003
6	-0.482	0.000	1.033	-1.033	0.002
7	0.089	3.000	2.810	0.190	0.000
8	-0.915	0.000	1.818	-1.818	0.035
9	-0.388	0.000	0.807	-0.807	0.003
10	-1.434	1.000	4.040	-3.040	0.026
11	1.936	6.000	1.842	4.158	0.027
12	0.489	5.000	3.982	1.018	0.005
13	-0.525	0.000	1.115	-1.115	0.003
14	-0.691	0.000	1.326	-1.326	0.028
15	1.075	3.000	0.736	2.264	0.017
16	-0.892	0.000	1.914	-1.914	0.006
17	0.105	5.000	4.791	0.209	0.000
18	0.465	4.000	3.029	0.971	0.004
19	-0.363	0.000	0.711	-0.711	0.006
20	0.042	4.000	3.911	0.089	0.000
21	-1.449	0.000	3.110	-3.110	0.015
22	0.300	3.000	2.352	0.648	0.000
23	-1.151	1.000	3.435	-2.435	0.017
24	-0.003	3.000	3.007	-0.007	0.000
25	1.313	7.000	4.239	2.761	0.028
26	0.099	3.000	2.791	0.209	0.000
27	-0.218	3.000	3.450	-0.450	0.001
28	-1.377	0.000	2.903	-2.903	0.028
29	-0.405	1.000	1.870	-0.870	0.001

Table S3. Casewise Diagnostics

Case Number	Std. Residual	Disfunción social	Predicted Value	Residual	Cook's Distance
30	0.489	2.000	0.969	1.031	0.003
31	-0.911	1.000	2.926	-1.926	0.011
32	-0.817	1.000	2.749	-1.749	0.006
33	1.620	7.000	3.631	3.369	0.054
34	0.860	6.000	4.205	1.795	0.014
35	0.362	3.000	2.234	0.766	0.002
36	-1.922	0.000	4.048	-4.048	0.057
37	-0.794	0.000	1.602	-1.602	0.022
38	-0.208	0.000	0.428	-0.428	0.001
39	-1.967	0.000	4.034	-4.034	0.105
40	-0.608	0.000	1.281	-1.281	0.006
41	-1.671	0.000	3.295	-3.295	0.127
42	-0.192	0.000	0.374	-0.374	0.002
43	1.041	6.000	3.803	2.197	0.015
44	-1.190	0.000	2.567	-2.567	0.007
45	0.244	2.000	1.480	0.520	0.001
46	-0.600	0.000	1.285	-1.285	0.003
47	-0.359	1.000	1.769	-0.769	0.001
48	0.396	3.000	2.157	0.843	0.002
49	0.659	2.000	0.653	1.347	0.012
50	0.122	1.000	0.751	0.249	0.000
51	0.539	2.000	0.890	1.110	0.007
52	1.324	6.000	3.170	2.830	0.016
53	1.811	7.000	3.167	3.833	0.043
54	0.906	5.000	3.091	1.909	0.012
55	-1.431	0.000	3.026	-3.026	0.028
56	1.901	7.000	2.993	4.007	0.054
57	1.811	7.000	3.167	3.833	0.043
58	1.583	5.000	1.671	3.329	0.040
59	-0.354	0.000	0.707	-0.707	0.005
60	-0.503	0.000	1.062	-1.062	0.004

Depression (GHQ)

Figure S7. Residuals vs. Predicted

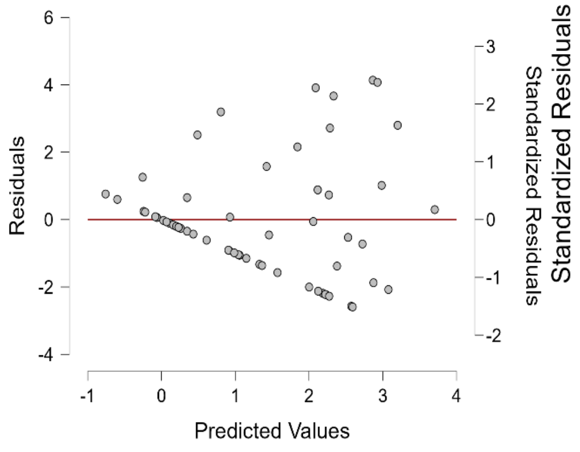

Figure S8. Q-Q Plot Standardized Residuals

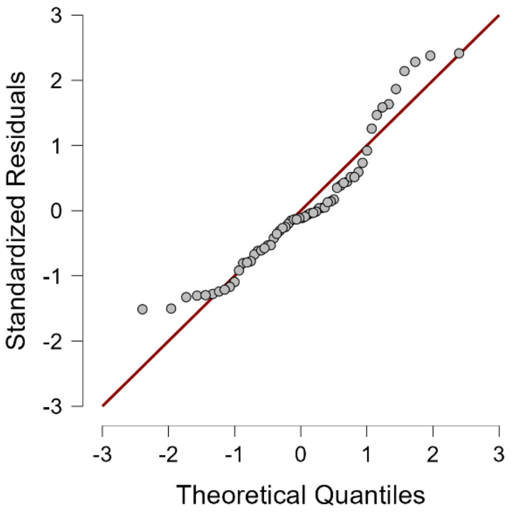

Table S4. Casewise Diagnostics

Case Number	Std. Residual	Depresión	Predicted Value	Residual	Cook's Distance
1	0.903	3.000	1.424	1.576	0.006
2	0.043	1.000	0.928	0.072	0.000
3	-0.064	0.000	0.109	-0.109	0.000
4	-0.151	0.000	0.261	-0.261	0.000
5	-0.611	0.000	1.057	-1.057	0.004
6	-0.112	0.000	0.195	-0.195	0.000
7	1.240	4.000	1.844	2.156	0.013
8	0.404	1.000	0.347	0.653	0.007
9	0.037	0.000	-0.062	0.062	0.000
10	-1.089	1.000	2.874	-1.874	0.015
11	1.832	4.000	0.805	3.195	0.024
12	1.653	6.000	3.202	2.798	0.054
13	-0.015	0.000	0.025	-0.025	0.000
14	-0.223	0.000	0.347	-0.347	0.003
15	-0.040	0.000	0.068	-0.068	0.000
16	-0.659	0.000	1.150	-1.150	0.003
17	0.183	4.000	3.705	0.295	0.001
18	-0.813	1.000	2.379	-1.379	0.013
19	-0.270	0.000	0.431	-0.431	0.004
20	-0.425	2.000	2.725	-0.725	0.003
21	-1.256	0.000	2.190	-2.190	0.012
22	-0.757	0.000	1.328	-1.328	0.003
23	-1.299	0.000	2.233	-2.233	0.022
24	2.185	6.000	2.333	3.667	0.113
25	0.594	4.000	2.985	1.015	0.006
26	-0.034	2.000	2.058	-0.058	0.000
27	-1.265	0.000	2.124	-2.124	0.038
28	-1.169	0.000	2.002	-2.002	0.020

Table S4. Casewise Diagnostics

Case Number	Std. Residual	Depresión	Predicted Value	Residual	Cook's Distance
29	-0.524	0.000	0.914	-0.914	0.002
30	-0.092	0.000	0.158	-0.158	0.000
31	-1.293	0.000	2.221	-2.221	0.023
32	-0.905	0.000	1.573	-1.573	0.007
33	2.447	7.000	2.865	4.135	0.124
34	2.402	7.000	2.928	4.072	0.111
35	-0.794	0.000	1.363	-1.363	0.009
36	-1.214	1.000	3.076	-2.076	0.023
37	-0.637	0.000	1.044	-1.044	0.014
38	0.454	0.000	-0.759	0.759	0.005
39	-1.545	0.000	2.573	-2.573	0.064
40	-0.117	0.000	0.200	-0.200	0.000
41	-1.618	0.000	2.591	-2.591	0.119
42	0.379	0.000	-0.599	0.599	0.008
43	-0.309	2.000	2.529	-0.529	0.001
44	-0.261	1.000	1.457	-0.457	0.000
45	-0.353	0.000	0.611	-0.611	0.001
46	-0.133	0.000	0.231	-0.231	0.000
47	-0.522	0.000	0.907	-0.907	0.002
48	-0.569	0.000	0.985	-0.985	0.003
49	0.051	0.000	-0.084	0.084	0.000
50	0.146	0.000	-0.243	0.243	0.001
51	0.751	1.000	-0.256	1.256	0.014
52	0.421	3.000	2.269	0.731	0.002
53	0.513	3.000	2.118	0.882	0.003
54	2.284	6.000	2.090	3.910	0.078
55	-1.324	0.000	2.273	-2.273	0.024
56	1.586	5.000	2.285	2.715	0.038
57	0.513	3.000	2.118	0.882	0.003
58	1.471	3.000	0.487	2.513	0.035
59	0.137	0.000	-0.222	0.222	0.001
60	-0.135	0.000	0.231	-0.231	0.000

Overall health (GHQ)

Figure S9. Residuals vs. Predicted

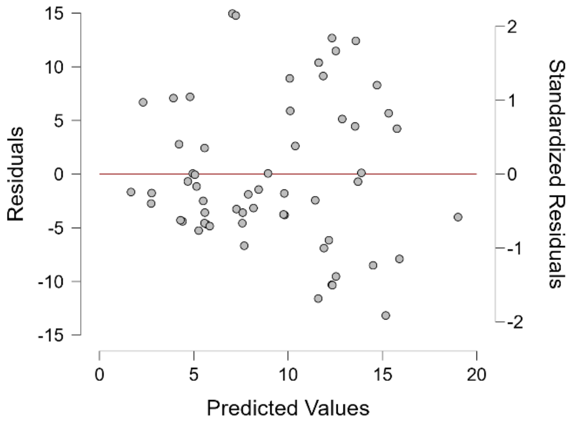

Figure S10. Q-Q Plot Standardized Residuals

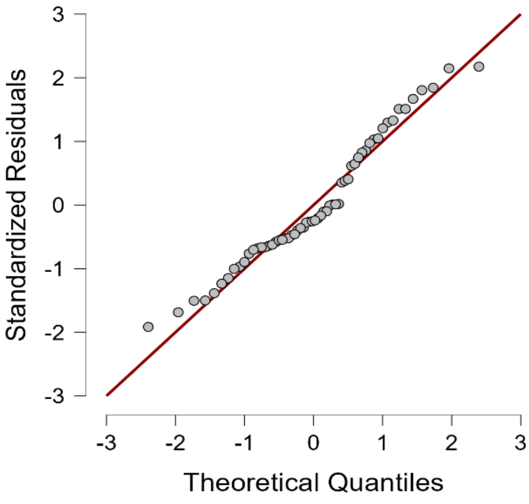

Table S5. Casewise Diagnostics

Case Number	Std. Residual	total GHQ	Predicted Value	Residual	Cook's Distance
1	1.272	19.000	10.087	8.913	0.011
2	-0.213	7.000	8.438	-1.438	0.001
3	0.357	8.000	5.573	2.427	0.003
4	-0.637	0.000	4.404	-4.404	0.005
5	-0.470	4.000	7.263	-3.263	0.002
6	0.007	5.000	4.948	0.052	0.000
7	0.843	16.000	10.112	5.888	0.006
8	-0.722	1.000	5.681	-4.681	0.022
9	-0.776	0.000	5.261	-5.261	0.013
10	-1.907	2.000	15.176	-13.176	0.046
11	2.136	22.000	7.048	14.952	0.032
12	0.622	20.000	15.773	4.227	0.008
13	-0.008	5.000	5.058	-0.058	0.000
14	-0.730	1.000	5.567	-4.567	0.032
15	1.031	11.000	3.921	7.079	0.016
16	-0.954	1.000	7.673	-6.673	0.007
17	-0.619	15.000	19.007	-4.007	0.016
18	-0.358	9.000	11.438	-2.438	0.002
19	-0.672	0.000	4.296	-4.296	0.022
20	0.017	14.000	13.885	0.115	0.000
21	-1.363	3.000	12.537	-9.537	0.014
22	0.009	9.000	8.936	0.064	0.000
23	-1.495	2.000	12.315	-10.315	0.029
24	1.357	21.000	11.862	9.138	0.044
25	0.827	21.000	15.331	5.669	0.011
26	0.380	13.000	10.382	2.618	0.002
27	-0.105	13.000	13.705	-0.705	0.000

Table S5. Casewise Diagnostics

Case Number	Std. Residual	total GHQ	Predicted Value	Residual	Cook's Distance
28	-1.004	5.000	11.900	-6.900	0.015
29	-0.513	4.000	7.593	-3.593	0.002
30	-0.521	2.000	5.587	-3.587	0.004
31	-0.894	6.000	12.163	-6.163	0.011
32	-0.547	6.000	9.814	-3.814	0.003
33	1.830	26.000	13.589	12.411	0.069
34	1.218	23.000	14.713	8.287	0.029
35	-0.261	8.000	9.798	-1.798	0.001
36	-1.151	8.000	15.903	-7.903	0.020
37	-0.287	6.000	7.888	-1.888	0.003
38	-0.408	0.000	2.739	-2.739	0.004
39	-1.272	6.000	14.505	-8.505	0.044
40	-0.099	4.000	4.683	-0.683	0.000
41	-1.610	2.000	12.348	-10.348	0.118
42	0.439	7.000	4.218	2.782	0.010
43	0.646	18.000	13.551	4.449	0.006
44	-0.534	6.000	9.759	-3.759	0.001
45	-0.360	3.000	5.498	-2.498	0.001
46	-0.165	4.000	5.150	-1.150	0.000
47	-0.454	5.000	8.165	-3.165	0.002
48	-0.658	3.000	7.574	-4.574	0.004
49	1.004	9.000	2.312	6.688	0.029
50	-0.265	1.000	2.767	-1.767	0.002
51	1.073	12.000	4.802	7.198	0.029
52	0.736	18.000	12.869	5.131	0.005
53	1.505	22.000	11.619	10.381	0.030
54	1.845	25.000	12.326	12.674	0.051
55	-1.682	0.000	11.596	-11.596	0.038
56	1.670	24.000	12.529	11.471	0.042
57	1.505	22.000	11.619	10.381	0.030
58	2.155	22.000	7.228	14.772	0.074
59	-0.256	0.000	1.671	-1.671	0.003
60	-0.705	1.000	5.848	-4.848	0.007

Self-esteem (RSES)

Figure S11. Residuals vs. Predicted

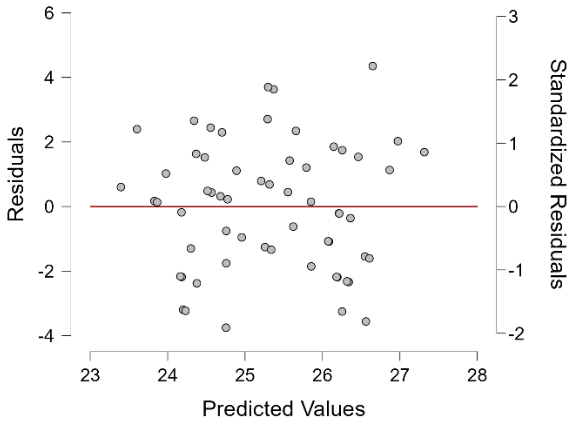

Figure S12. Q-Q Plot Standardized Residuals

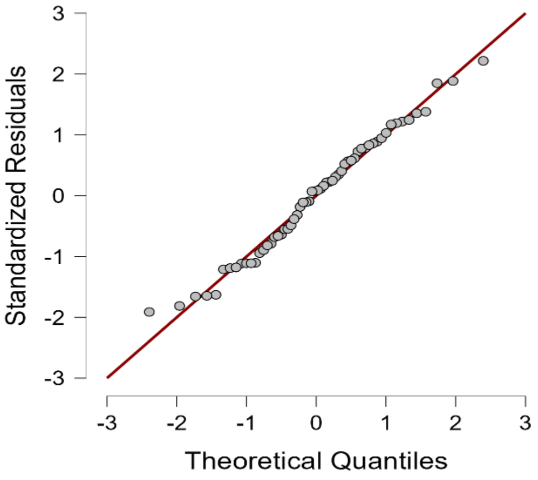

Table S6. Casewise Diagnostics

Case Number	Std. Residual	Autoestima	Predicted Value	Residual	Cook's Distance
1	-0.629	24.000	25.257	-1.257	0.003
2	-0.563	25.000	26.083	-1.083	0.007
3	-1.679	23.000	26.253	-3.253	0.057
4	-0.544	25.000	26.074	-1.074	0.004
5	0.719	27.000	25.576	1.424	0.005
6	-1.099	24.000	26.191	-2.191	0.010
7	0.557	26.000	24.889	1.111	0.003
8	-0.837	25.000	26.548	-1.548	0.029
9	0.795	28.000	26.462	1.538	0.013
10	-1.107	22.000	24.182	-2.182	0.015
11	0.075	26.000	25.850	0.150	0.000
12	1.236	26.000	23.604	2.396	0.030
13	-1.800	23.000	26.561	-3.561	0.036
14	0.977	28.000	26.256	1.744	0.057
15	0.946	28.000	26.145	1.855	0.014
16	0.343	26.000	25.316	0.684	0.001
17	0.327	24.000	23.397	0.603	0.005
18	-0.092	24.000	24.179	-0.179	0.000
19	0.244	26.000	25.554	0.446	0.003
20	1.360	27.000	24.342	2.658	0.030
21	0.216	25.000	24.568	0.432	0.000
22	1.808	29.000	25.369	3.631	0.016
23	0.114	25.000	24.775	0.225	0.000
24	-1.664	21.000	24.199	-3.199	0.066
25	-1.107	22.000	24.165	-2.165	0.020
26	0.246	25.000	24.516	0.484	0.001
27	-0.498	24.000	24.957	-0.957	0.006

Table S6. Casewise Diagnostics

Case Number	Std. Residual	Autoestima	Predicted Value	Residual	Cook's Distance
28	0.162	25.000	24.682	0.318	0.000
29	1.173	28.000	25.657	2.343	0.010
30	-0.108	26.000	26.212	-0.212	0.000
31	-1.208	22.000	24.377	-2.377	0.020
32	1.858	29.000	25.299	3.701	0.029
33	0.088	24.000	23.829	0.171	0.000
34	-1.664	21.000	24.231	-3.231	0.053
35	0.403	26.000	25.208	0.792	0.002
36	0.069	24.000	23.865	0.135	0.000
37	1.443	28.000	25.291	2.709	0.072
38	0.880	29.000	27.315	1.685	0.020
39	1.204	27.000	24.703	2.297	0.039
40	-0.184	26.000	26.361	-0.361	0.001
41	0.557	25.000	23.979	1.021	0.014
42	1.120	29.000	26.973	2.027	0.066
43	1.245	27.000	24.554	2.446	0.022
44	-0.666	24.000	25.337	-1.337	0.002
45	-0.936	24.000	25.855	-1.855	0.009
46	-1.173	24.000	26.339	-2.339	0.011
47	-0.313	25.000	25.623	-0.623	0.001
48	0.609	27.000	25.792	1.208	0.004
49	-1.219	24.000	26.317	-2.317	0.043
50	2.286	31.000	26.648	4.352	0.147
51	0.591	28.000	26.868	1.132	0.009
52	0.764	26.000	24.479	1.521	0.005
53	-0.892	23.000	24.757	-1.757	0.011
54	-1.914	21.000	24.753	-3.753	0.055
55	0.829	26.000	24.368	1.632	0.009
56	-0.663	23.000	24.300	-1.300	0.007
57	-0.384	24.000	24.757	-0.757	0.002
58	-0.111	26.000	26.216	-0.216	0.000
59	-0.863	25.000	26.606	-1.606	0.029
60	-1.111	24.000	26.182	-2.182	0.018

Positive stress (PSS)

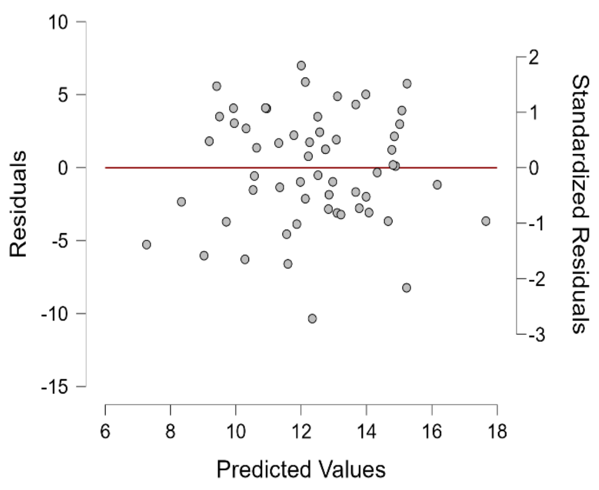

Figure S13. Residuals vs. Predicted

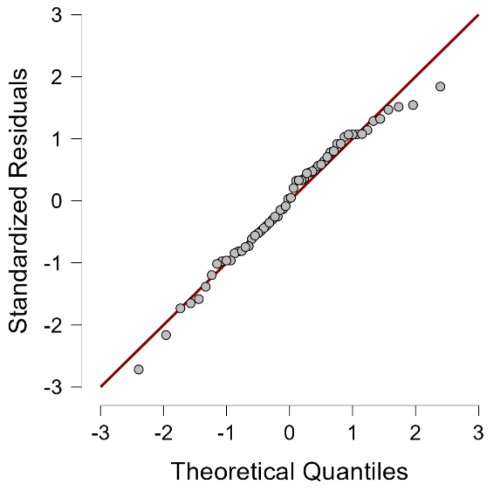

Figure S14. Q-Q Plot Standardized Residuals

Table S7. Casewise Diagnostics

Case Number	Std. Residual	Factor positivo	Predicted Value	Residual	Cook's Distance
1	0.450	14.000	12.257	1.743	0.001
2	-0.832	10.000	13.100	-3.100	0.016
3	-1.755	5.000	11.593	-6.593	0.062
4	-0.968	6.000	9.706	-3.706	0.011
5	-2.693	2.000	12.340	-10.340	0.076
6	0.353	12.000	10.634	1.366	0.001
7	-0.480	11.000	12.852	-1.852	0.002
8	-0.897	10.000	13.217	-3.217	0.034
9	1.087	14.000	9.925	4.075	0.025
10	-2.153	7.000	15.227	-8.227	0.058
11	1.051	15.000	10.932	4.068	0.008
12	-0.818	11.000	14.074	-3.074	0.013
13	-1.638	4.000	10.278	-6.278	0.030
14	-1.522	2.000	7.266	-5.266	0.138
15	-0.402	9.000	10.526	-1.526	0.002
16	-1.177	7.000	11.554	-4.554	0.010
17	-1.022	14.000	17.657	-3.657	0.044
18	0.646	15.000	12.567	2.433	0.008
19	-0.660	6.000	8.334	-2.334	0.021
20	-0.733	11.000	13.777	-2.777	0.009
21	-0.431	12.000	13.668	-1.668	0.001
22	-0.249	12.000	12.968	-0.968	0.000
23	0.504	15.000	13.077	1.923	0.003
24	0.576	17.000	14.852	2.148	0.008
25	1.033	19.000	15.081	3.919	0.017
26	-0.135	12.000	12.515	-0.515	0.000
27	0.030	15.000	14.889	0.111	0.000
28	0.049	15.000	14.812	0.188	0.000
29	0.325	14.000	12.742	1.258	0.001

Table S7. Casewise Diagnostics

Case Number	Std. Residual	Factor positivo	Predicted Value	Residual	Cook's Distance
30	-0.149	10.000	10.567	-0.567	0.000
31	0.916	16.000	12.505	3.495	0.011
32	-0.734	10.000	12.835	-2.835	0.005
33	1.535	21.000	15.241	5.759	0.049
34	0.325	16.000	14.776	1.224	0.002
35	1.136	18.000	13.670	4.330	0.018
36	-0.965	11.000	14.662	-3.662	0.014
37	-1.656	3.000	9.025	-6.025	0.095
38	0.489	11.000	9.186	1.814	0.006
39	-0.089	14.000	14.327	-0.327	0.000
40	-1.017	8.000	11.865	-3.865	0.016
41	-0.328	15.000	16.168	-1.168	0.005
42	-0.384	10.000	11.345	-1.345	0.008
43	0.783	18.000	15.017	2.983	0.009
44	1.256	18.000	13.110	4.890	0.008
45	1.057	15.000	10.944	4.056	0.012
46	1.060	15.000	10.907	4.093	0.009
47	0.437	13.000	11.313	1.687	0.002
48	-0.254	11.000	11.975	-0.975	0.001
49	0.729	13.000	10.313	2.687	0.015
50	0.603	14.000	11.776	2.224	0.010
51	1.506	15.000	9.412	5.588	0.058
52	1.302	19.000	13.978	5.022	0.015
53	1.539	18.000	12.128	5.872	0.031
54	1.841	19.000	12.002	6.998	0.051
55	0.205	13.000	12.219	0.781	0.001
56	-0.523	12.000	13.989	-1.989	0.004
57	-0.558	10.000	12.128	-2.128	0.004
58	0.331	14.000	12.746	1.254	0.002
59	0.971	13.000	9.499	3.501	0.037
60	0.802	13.000	9.951	3.049	0.009

Negative stress (PSS)

Figure S15. Residuals vs. Predicted

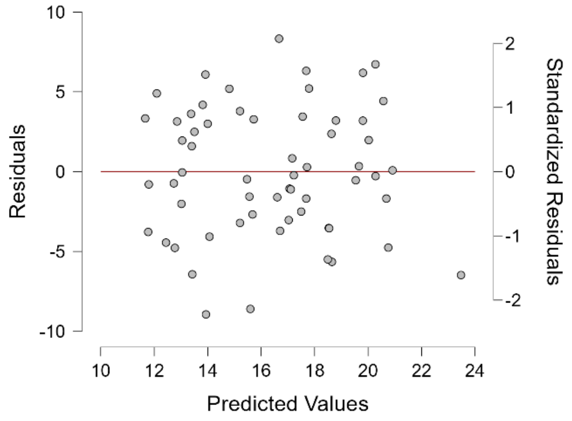

Figure S16. Q-Q Plot Standardized Residuals

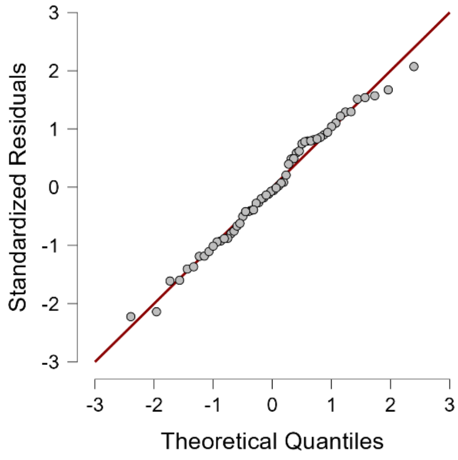

Table S8. Casewise Diagnostics

Case Number	Std. Residual	Factor negativo	Predicted Value	Residual	Cook's Distance
1	2.033	25.000	16.672	8.328	0.028
2	-0.941	13.000	16.709	-3.709	0.021
3	1.532	20.000	13.919	6.081	0.047
4	-1.180	8.000	12.775	-4.775	0.017
5	-2.118	7.000	15.598	-8.598	0.047
6	0.886	17.000	13.381	3.619	0.006
7	-0.055	17.000	17.224	-0.224	0.000
8	-0.125	15.000	15.473	-0.473	0.001
9	0.492	15.000	13.050	1.950	0.005
10	-1.177	16.000	20.757	-4.757	0.017
11	1.267	20.000	14.812	5.188	0.011
12	1.692	27.000	20.277	6.723	0.057
13	-1.586	7.000	13.427	-6.427	0.028
14	-1.215	8.000	12.443	-4.443	0.088
15	-0.183	12.000	12.735	-0.735	0.001
16	-0.784	12.000	15.210	-3.210	0.004
17	-1.712	17.000	23.478	-6.478	0.125
18	0.866	21.000	17.554	3.446	0.014
19	-1.010	8.000	11.776	-3.776	0.049
20	0.085	20.000	19.661	0.339	0.000
21	-0.863	15.000	18.531	-3.531	0.005
22	-0.390	15.000	16.605	-1.605	0.001
23	-0.879	15.000	18.547	-3.547	0.010
24	0.601	21.000	18.632	2.368	0.009
25	0.021	21.000	20.917	0.083	0.000
26	-0.264	16.000	17.064	-1.064	0.001
27	0.810	23.000	19.807	3.193	0.015
28	-1.406	13.000	18.650	-5.650	0.029

Table S8. Casewise Diagnostics

Case Number	Std. Residual	Factor negativo	Predicted Value	Residual	Cook's Distance
29	0.801	19.000	15.723	3.277	0.005
30	0.620	16.000	13.507	2.493	0.005
31	0.070	18.000	17.716	0.284	0.000
32	0.205	18.000	17.164	0.836	0.000
33	1.561	26.000	19.811	6.189	0.050
34	1.112	25.000	20.577	4.423	0.024
35	-0.753	14.000	17.033	-3.033	0.008
36	-0.420	19.000	20.685	-1.685	0.003
37	-2.324	5.000	13.939	-8.939	0.187
38	-0.204	11.000	11.800	-0.800	0.001
39	-0.072	20.000	20.282	-0.282	0.000
40	-1.013	10.000	14.071	-4.071	0.015
41	-0.144	19.000	19.541	-0.541	0.001
42	-0.013	13.000	13.049	-0.049	0.000
43	0.490	22.000	20.027	1.973	0.003
44	-0.270	16.000	17.110	-1.110	0.000
45	0.739	17.000	14.002	2.998	0.006
46	1.024	18.000	13.817	4.183	0.008
47	0.929	19.000	15.210	3.790	0.008
48	-0.660	13.000	15.680	-2.680	0.004
49	1.259	17.000	12.098	4.902	0.045
50	-0.518	11.000	13.020	-2.020	0.008
51	0.801	16.000	12.856	3.144	0.016
52	0.786	22.000	18.798	3.202	0.005
53	1.565	24.000	17.686	6.314	0.032
54	1.296	23.000	17.792	5.208	0.025
55	-0.620	15.000	17.501	-2.501	0.005
56	-1.368	13.000	18.496	-5.496	0.028
57	-0.418	16.000	17.686	-1.686	0.002
58	-0.390	14.000	15.564	-1.564	0.002
59	0.875	15.000	11.667	3.333	0.030
60	0.396	15.000	13.407	1.593	0.002

Positive care appreciation (ECI)

Figure S17. Residuals vs. Predicted

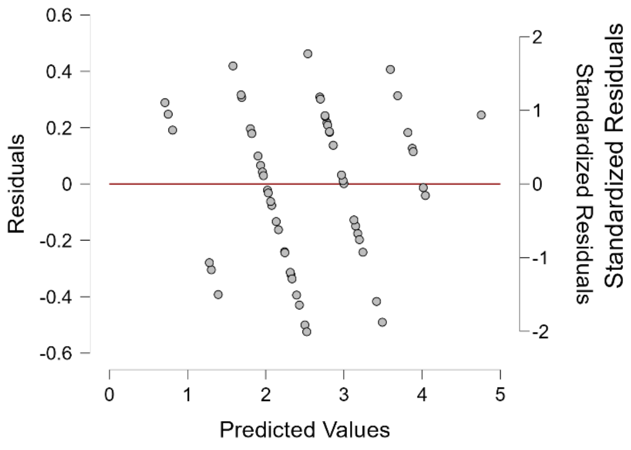

Figure S18. Q-Q Plot Standardized Residuals

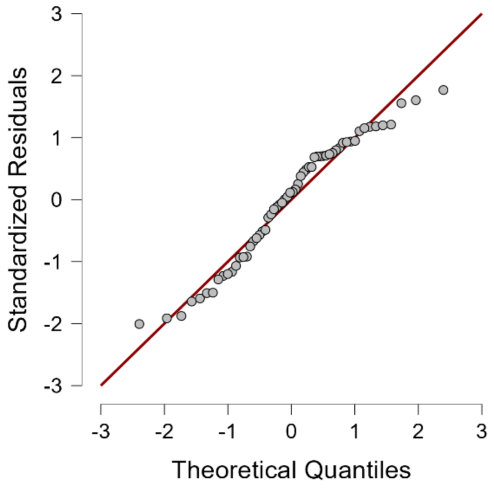

Table S9. Casewise Diagnostics

Case Number	Std. Residual	Aspec posti media	Predicted Value	Residual	Cook's Distance
1	0.689	3.000	2.817	0.183	0.003
2	0.005	3.000	2.999	0.001	0.000
3	-0.294	2.000	2.076	-0.076	0.002
4	-1.158	1.000	1.305	-0.305	0.016
5	-0.235	2.000	2.062	-0.062	0.001
6	1.157	2.000	1.693	0.307	0.011
7	-1.885	2.000	2.500	-0.500	0.029
8	-1.744	2.000	2.430	-0.430	0.127
9	-0.085	2.000	2.022	-0.022	0.000
10	0.482	4.000	3.873	0.127	0.003
11	-0.502	2.000	2.133	-0.133	0.002
12	-1.899	3.000	3.491	-0.491	0.072
13	-0.121	2.000	2.032	-0.032	0.000
14	1.330	2.000	1.684	0.316	0.105
15	-1.070	1.000	1.279	-0.279	0.017
16	0.248	2.000	1.934	0.066	0.000
17	0.996	5.000	4.755	0.245	0.042
18	-1.243	2.000	2.322	-0.322	0.029
19	1.187	1.000	0.711	0.289	0.068
20	-1.601	3.000	3.417	-0.417	0.042
21	-0.660	3.000	3.176	-0.176	0.003
22	1.728	3.000	2.538	0.462	0.015
23	-0.753	3.000	3.197	-0.197	0.007
24	1.206	3.000	2.691	0.309	0.034
25	0.440	4.000	3.885	0.115	0.003
26	-0.918	2.000	2.240	-0.240	0.012
27	-0.160	4.000	4.041	-0.041	0.001

Table S9. Casewise Diagnostics

Case Number	Std. Residual	Aspec posti media	Predicted Value	Residual	Cook's Distance
28	-0.571	3.000	3.149	-0.149	0.005
29	-1.179	2.000	2.314	-0.314	0.010
30	0.163	2.000	1.957	0.043	0.000
31	0.829	3.000	2.783	0.217	0.009
32	0.903	3.000	2.761	0.239	0.007
33	0.046	3.000	2.988	0.012	0.000
34	1.212	4.000	3.687	0.313	0.028
35	0.796	3.000	2.792	0.208	0.009
36	0.700	4.000	3.817	0.183	0.008
37	0.729	2.000	1.818	0.182	0.018
38	0.769	2.000	1.804	0.196	0.015
39	-0.052	4.000	4.013	-0.013	0.000
40	0.685	2.000	1.821	0.179	0.007
41	0.992	3.000	2.758	0.242	0.045
42	-1.013	2.000	2.244	-0.244	0.054
43	1.554	4.000	3.593	0.407	0.034
44	0.695	3.000	2.814	0.186	0.002
45	1.589	2.000	1.581	0.419	0.027
46	0.374	2.000	1.901	0.099	0.001
47	-1.487	2.000	2.394	-0.394	0.019
48	-1.274	2.000	2.337	-0.337	0.017
49	0.755	1.000	0.809	0.191	0.016
50	-1.547	1.000	1.392	-0.392	0.067
51	-0.637	2.000	2.162	-0.162	0.010
52	-0.914	3.000	3.242	-0.242	0.007
53	0.524	3.000	2.863	0.137	0.004
54	-0.489	3.000	3.128	-0.128	0.004
55	-2.001	2.000	2.524	-0.524	0.054
56	0.123	3.000	2.968	0.032	0.000
57	0.524	3.000	2.863	0.137	0.004
58	1.156	3.000	2.699	0.301	0.021
59	1.000	1.000	0.752	0.248	0.039
60	0.113	2.000	1.970	0.030	0.000

Negative care appreciation (ECI)

Figure S19. Residuals vs. Predicted

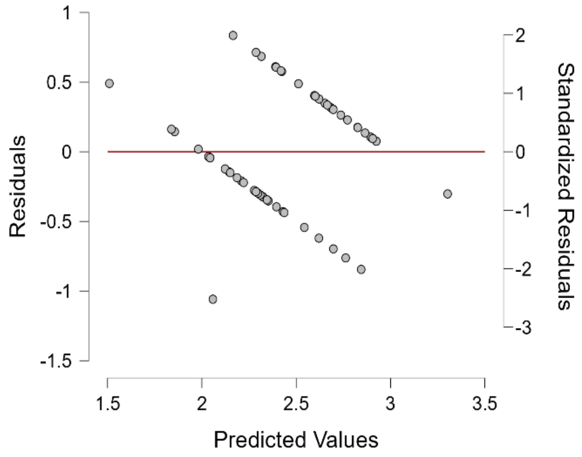

Figure S20. Q-Q Plot Standardized Residuals

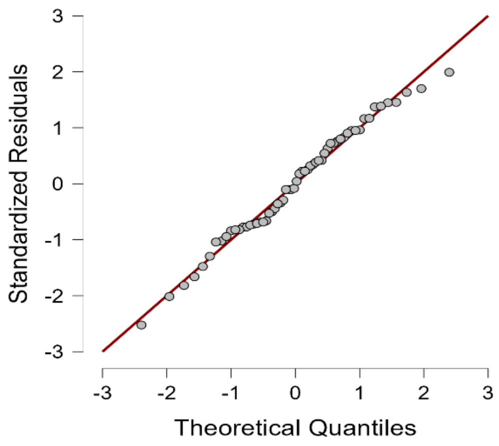

Table S10. Casewise Diagnostics

Case Number	Std. Residual	Apsct negat media	Predicted Value	Residual	Cook's Distance
1	0.742	3.000	2.683	0.317	0.004
2	-0.351	2.000	2.144	-0.144	0.003
3	0.636	3.000	2.737	0.263	0.008
4	-0.292	2.000	2.123	-0.123	0.001
5	-2.499	1.000	2.058	-1.058	0.066
6	-0.828	2.000	2.353	-0.353	0.006
7	-0.492	2.000	2.210	-0.210	0.002
8	0.364	2.000	1.856	0.144	0.006
9	0.737	3.000	2.696	0.304	0.011
10	-1.655	2.000	2.697	-0.697	0.034
11	1.955	3.000	2.165	0.835	0.027
12	0.253	3.000	2.895	0.105	0.001
13	-0.680	2.000	2.287	-0.287	0.005
14	-0.114	2.000	2.043	-0.043	0.001
15	-0.774	2.000	2.324	-0.324	0.009
16	-0.757	2.000	2.323	-0.323	0.004
17	-0.767	3.000	3.303	-0.303	0.025
18	1.469	3.000	2.390	0.610	0.041
19	-0.883	2.000	2.344	-0.344	0.038
20	-0.740	2.000	2.309	-0.309	0.009
21	-1.787	2.000	2.762	-0.762	0.023
22	-0.804	2.000	2.345	-0.345	0.003
23	-0.660	2.000	2.278	-0.278	0.006
24	0.738	3.000	2.697	0.303	0.013
25	1.167	3.000	2.512	0.488	0.022
26	-0.708	2.000	2.297	-0.297	0.007
27	0.328	3.000	2.865	0.135	0.003
28	-2.015	2.000	2.845	-0.845	0.060

Table S10. Casewise Diagnostics

Case Number	Std. Residual	Apsct negat media	Predicted Value	Residual	Cook's Distance
29	1.350	3.000	2.424	0.576	0.013
30	0.829	3.000	2.652	0.348	0.010
31	0.543	3.000	2.772	0.228	0.004
32	-0.437	2.000	2.186	-0.186	0.002
33	0.975	3.000	2.597	0.403	0.020
34	1.649	3.000	2.316	0.684	0.052
35	0.416	3.000	2.825	0.175	0.002
36	0.225	3.000	2.906	0.094	0.001
37	-1.546	2.000	2.620	-0.620	0.083
38	-0.541	2.000	2.221	-0.221	0.008
39	-1.055	2.000	2.430	-0.430	0.030
40	-0.104	2.000	2.043	-0.043	0.000
41	1.014	3.000	2.602	0.398	0.047
42	0.195	3.000	2.925	0.075	0.002
43	1.446	3.000	2.393	0.607	0.030
44	-0.921	2.000	2.395	-0.395	0.004
45	-0.083	2.000	2.035	-0.035	0.000
46	-0.353	2.000	2.150	-0.150	0.001
47	0.789	3.000	2.665	0.335	0.005
48	-0.103	2.000	2.044	-0.044	0.000
49	0.397	2.000	1.839	0.161	0.005
50	0.046	2.000	1.981	0.019	0.000
51	1.420	3.000	2.419	0.581	0.051
52	0.409	3.000	2.826	0.174	0.001
53	1.695	3.000	2.287	0.713	0.038
54	0.904	3.000	2.621	0.379	0.012
55	-1.038	2.000	2.437	-0.437	0.015
56	0.226	3.000	2.905	0.095	0.001
57	-0.682	2.000	2.287	-0.287	0.006
58	-1.300	2.000	2.543	-0.543	0.027
59	1.233	2.000	1.510	0.490	0.059
60	0.951	3.000	2.601	0.399	0.013
